# Practical Synthesis of 6-Amino-1-hydroxy-2,1-benzoxaborolane: A Key Intermediate of DNDI-6148

**DOI:** 10.1021/acs.oprd.4c00031

**Published:** 2024-03-29

**Authors:** Pankaj V. Khairnar, John M. Saathoff, Daniel W. Cook, Samuel R. Hochstetler, Urvish Pandya, Stephen J. Robinson, Vijay Satam, Kai O. Donsbach, B. Frank Gupton, Li-Mei Jin, Charles S. Shanahan

**Affiliations:** † Medicines for All Institute, Virginia Commonwealth University, Richmond, Virginia 23284-3068, United States; ‡ Drugs for Neglected Diseases initiative, 15 Chemin Camille-Vidart, 1202 Geneva, Switzerland

**Keywords:** DNDI-6148, benzoxaborole, Hofmann rearrangement, diazotization, continuous flow trans-hydrogenation, nitration-free

## Abstract

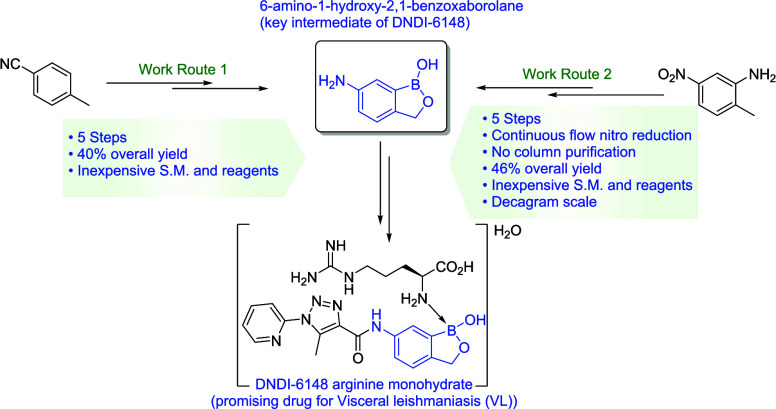

Visceral leishmaniasis (VL), a parasitic, poverty-linked, neglected disease, is endemic across multiple regions of the world and fatal if untreated. There is an urgent need for a better and more affordable treatment for VL. DNDI-6148 is a promising drug candidate being evaluated for the treatment of VL; however, the current process for producing the key intermediate of DNDI-6148, 6-amino-1-hydroxy-2,1-benzoxaborolane, is expensive and difficult to scale up. Herein, we describe two practical approaches to synthesizing 6-amino-1-hydroxy-2,1-benzoxaborolane from inexpensive and readily available raw materials. Starting with 4-tolunitrile, the first approach is a five-step sequence involving a Hofmann rearrangement, resulting in an overall yield of 40%. The second approach utilizes 2-methyl-5-nitroaniline as the starting material and features borylation of aniline and continuous flow hydrogenation as the key steps, with an overall yield of 46%. Both routes bypass the nitration of 1-hydroxy-2,1-benzoxaborolane, which is challenging and expensive to scale. In particular, the second approach is more practical and scalable because of the mild operating conditions and facile isolation process.

## Introduction

**Figure 1:**
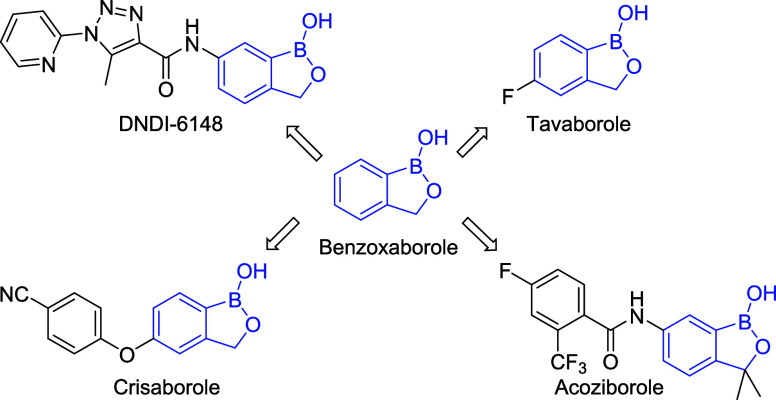
Biologically active molecules with a benzoxaborole core.

Leishmaniases are a group of poverty-linked diseases caused by the protozoa parasite *Leishmania*.^1,2^ Over 20 *Leishmania* species known to be infectious to humans are transmitted by the bite of infected female phlebotomine sand flies.^3^ Of the three main types of leishmaniasis, visceral leishmaniasis (VL) is the most severe form of the disease,^4,5^ with an estimated 50,000–90,000 cases reported each year.^6,7^ VL is characterized by irregular bouts of fever, weight loss, hepatosplenomegaly, and anemia. It can cause severe morbidity and death if left untreated.^8−11^ Current treatment options for this disease include pentavalent antimonials, amphotericin B, miltefosine, and paromomycin.^12^ However, these treatments have many drawbacks, such as parenteral administration, long treatment duration, severe toxicity, high cost, and emerging drug resistance.^13−16^ Thus, there is an urgent need to develop novel, less toxic, and low-cost oral treatments to combat this deadly, neglected disease. Benzoxaboroles are a versatile class of boron-heterocycles that are gaining attention as potential drug candidates due to their impressive biological activities, including antifungal, antibacterial, antiviral, anti-inflammatory, and antiprotozoal properties.^16−19^ This breadth of activities is attributed to their extraordinary physical and chemical properties.^20,21^ Two benzoxaborole derivatives, tavaborole and crisaborole, are already used for the treatment of onychomycosis (tavaborole) and atopic dermatitis (crisaborole), with several others in various phases of clinical development (Figure 1).^22−28^

In an attempt to develop new, affordable treatments for VL, the Drugs for Neglected Diseases initiative (DNDi) screened a library of compounds from the benzoxaborole (1-hydroxy-2,1-benzoxaborolane) class. Subsequently, a lead optimization program led to the identification of DNDI-6148 (Figure 1). The *in vitro* and *in vivo* studies confirmed an impressive activity of DNDI-6148 against the *Leishmania* strains^28−31^ and paved the way for its evaluation in healthy human volunteers in a Phase I clinical trial.^29^

**Scheme 1:**
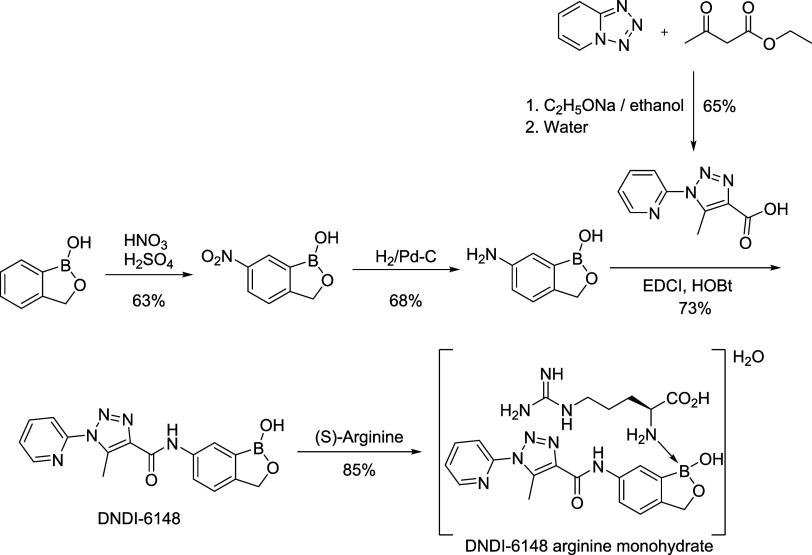
Current Synthetic Route for the Preparation of DNDI-6148 Arginine Monohydrate

The initial routes^31^ for producing DNDI-6148 have been developed to yield the current synthetic route shown in Scheme 1. From the drug development process, an arginine monohydrate adduct of the active substance DNDI-6148 has been identified as the chosen form (the final step in Scheme 1). The convergent synthesis involves two key intermediates, 6-amino-1-hydroxy-2,1-benzoxaborolane and 5-methyl-1-(pyridine-2-yl)1*H*-1,2,3-triazole-4-carboxylic acid. 6-Amino-1-hydroxy-2,1-benzoxaborolane is synthesized in two steps from 1-hydroxy-2,1-benzoxaborolane through nitration, followed by hydrogenation. 5-Methyl-1-(pyridine-2-yl)1*H*-1,2,3-triazole-4-carboxylic acid is synthesized in a one-pot reaction between tetrazolo[1,5-*a*]pyridine and ethyl acetoacetate under basic conditions, which sets the triazole ring, and subsequent hydrolysis of the intermediate ethyl ester generates the desired carboxylic acid. An amide bond formation between these two key intermediates affords the DNDI-6148 free acid, which is then converted to the crystalline arginine monohydrate adduct by reaction with (*S*)-arginine.

**Figure 2:**
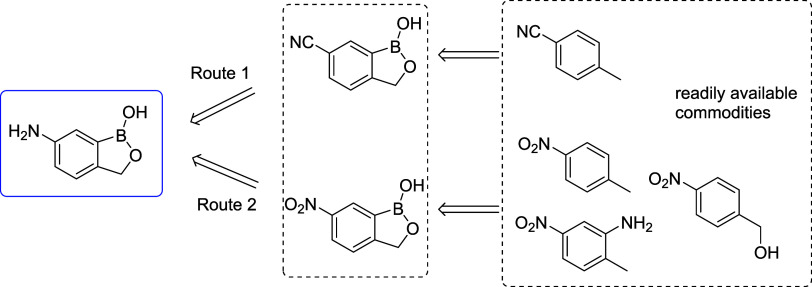
Key ideas for preparing 6-amino-1-hydroxy-2,1-benzoxaborolane from readily available cyano- or nitroaromatics.

Scheme 1 provides a concise and convergent route to prepare DNDI-6148; however, 1-hydroxy-2,1-benzoxaborolane is an expensive raw material. The original route to synthesize 6-amino-1-hydroxy-2,1-benzoxaborolane is via nitration and classical Clemmensen-type reduction of the nitro group.^32^ However, nitration of 1-hydroxy-2,1-benzoxaborolane has been found to be challenging to scale up primarily due to process safety, solubility, and stability of 1-hydroxy-2,1-benzoxaborolane in the nitration medium. The reduction of 6-nitro-1-hydroxy-2,1-benzoxaborolane has been further developed to replace the Clemmensen-type reduction with Pd–C-catalyzed hydrogenation, where the use of hydrogen gas could be prohibitive due to additional safety issues. In order to ensure broad, affordable access of DNDI-6148 to the patients suffering from VL in vulnerable communities, a safer, low-cost, and more practical process is required to prepare 6-amino-1-hydroxy-2,1-benzoxaborolane, one of the key cost drivers of DNDI-6148 API. To that end, in the present work, we aimed to mitigate these prior drawbacks by starting directly from a nitrile- or nitro-functionalized 1-hydroxy-2,1-benzoxaborolane (Figure 2). It is acknowledged that by starting with nitrated raw materials, we are not eliminating nitration; however, the chosen nitrated raw materials are inexpensive and readily available, and use an established, scalable nitration process.

## Results and Discussion

**Scheme 2:**
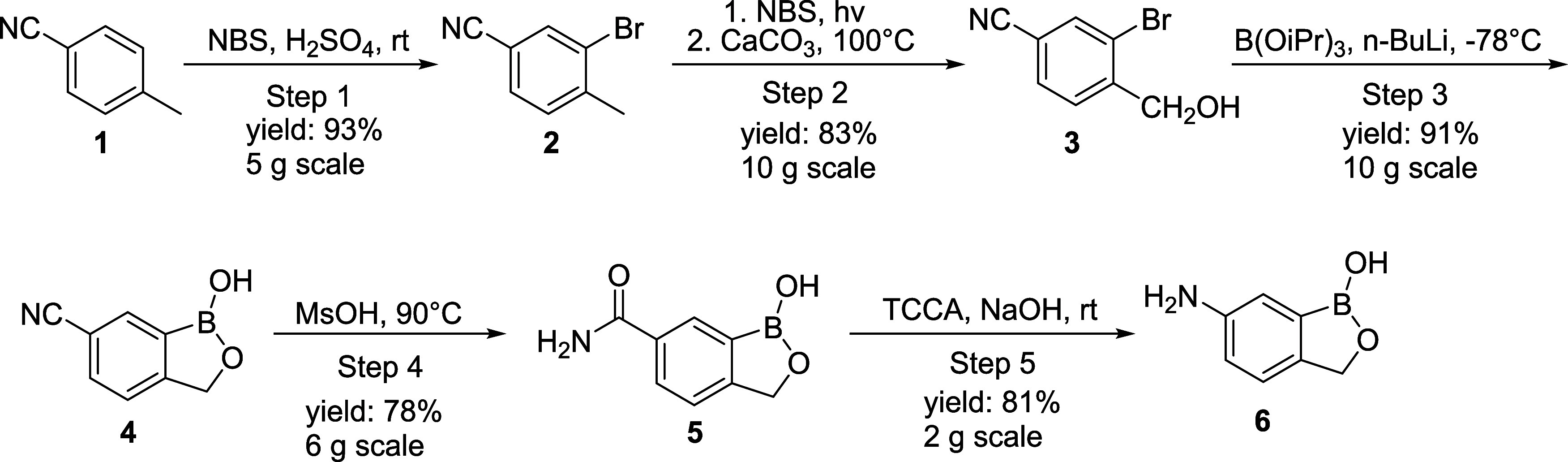
Synthetic Route for the Preparation of 6-Amino-1-hydroxy-2,1-benzoxaborolane (**6**) from 4-Tolunitrile (**1**)

Initial efforts to synthesize 6-amino-1-hydroxy-2,1-benzoxaborolane from 4-tolunitrile (Scheme 2) relied on mostly precedented transformations, but the final Hofmann transformation was unprecedented and was seen as pivotal to the success of the route.

We prepared the key intermediate 6-cyano-1-hydroxy-2,1-benzoxaborolane **4** according to the reported protocol (Scheme 2).^33^ Starting from commercially available 4-tolunitrile **1**, compound **4** was obtained in 70% overall yield. Converting the nitrile moiety **4** to the corresponding amide **5** is known to be problematic,^34^ suffering from a very low yield (15%) when treated with conc. H_2_SO_4_. To improve upon this, we screened a variety of acids and found methanesulfonic acid (MsOH) to be the optimal acid for this reaction, giving amide **5** in a 75% yield. We also explored basic nitrile hydrolysis and found KOH to provide the best conversion (∼60 LC area% of the product in the crude reaction mixture), but purification of the resulting amide failed due to overhydrolysis of the cyano group to carboxylic acid under the basic conditions.

With intermediate **5** in hand, the Hofmann rearrangement was investigated (Table 1). As shown in Table 1, commonly used oxidants were tested, i.e., NaOCl and Br_2_, for the Hofmann rearrangement.^35,36^ No product was formed when NaOCl was used. Br_2_, on the other hand, showed that ∼8 A% of the desired product was detected via LCMS; however, further optimization resulted in no improvement (Table 1, entries 1 and 2). Our study found TCCA (trichloroisocyanuric acid) to be effective in delivering the desired amine at 75 °C (55 A% by HPLC). Upon further studies, it was identified that 25 °C was the optimal temperature to give the maximum amount of the desired product (60–96 A% by HPLC, depending on the scale). Under the optimized conditions, up to 81% isolated yield was obtained on a gram scale (Table 1, entries 3–7). The reliability of this five-step sequence was successfully validated with decagram-scale reactions, and similar results were obtained. This route affords 6-amino-1-hydroxy-2,1-benzoxaborolane in 40% overall yield from readily available 4-tolunitrile and avoids the expensive 1-hydroxy-2,1-benzoxaborolane starting material and a hazardous nitration step. This route exhibits promise for scale-up; however, further optimization is required (i.e., eliminating the need for column chromatography).

**Table 1:**
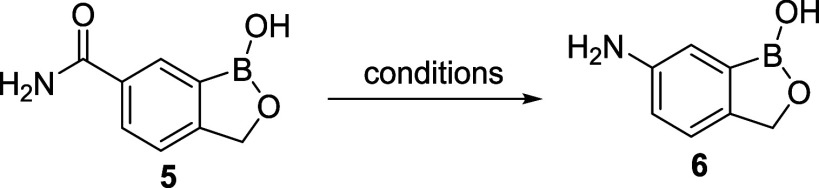
Hofmann Rearrangement of Amide **5** to Afford Amine **6**^a^

entry	conditions	A% (yield)^b^ of **6**
1	NaOH, NaOCl, 100 °C, 16 h	ND
2	NaOH, Br_2_, 75 °C, 12 h	8 (--c)
3	NaOH, TCCA, 75 °C, 12 h	55 (--c)
4	NaOH, TCCA, 55 °C, 12 h	57 (--c)
5	NaOH, TCCA, 25 °C, 12 h	60 (--c)
6	NaOH, TCCA, 25 °C, 12 h	78 (58)^d^
7	NaOH, TCCA, 25 °C, 12 h	96 (81)^e^

a All reactions were performed with **5** (50 mg, 1 equiv), oxidant (1 equiv), and temperature and reaction time as shown in the table unless otherwise stated; for TCCA, 0.35 eq was used.

b Area percentage under 210 nm unless otherwise stated and isolated yield in parentheses.

c No isolation.

d 100 mg of **5** was used.

e 2 g of **5** was used. ND: not detected.

**Scheme 3:**
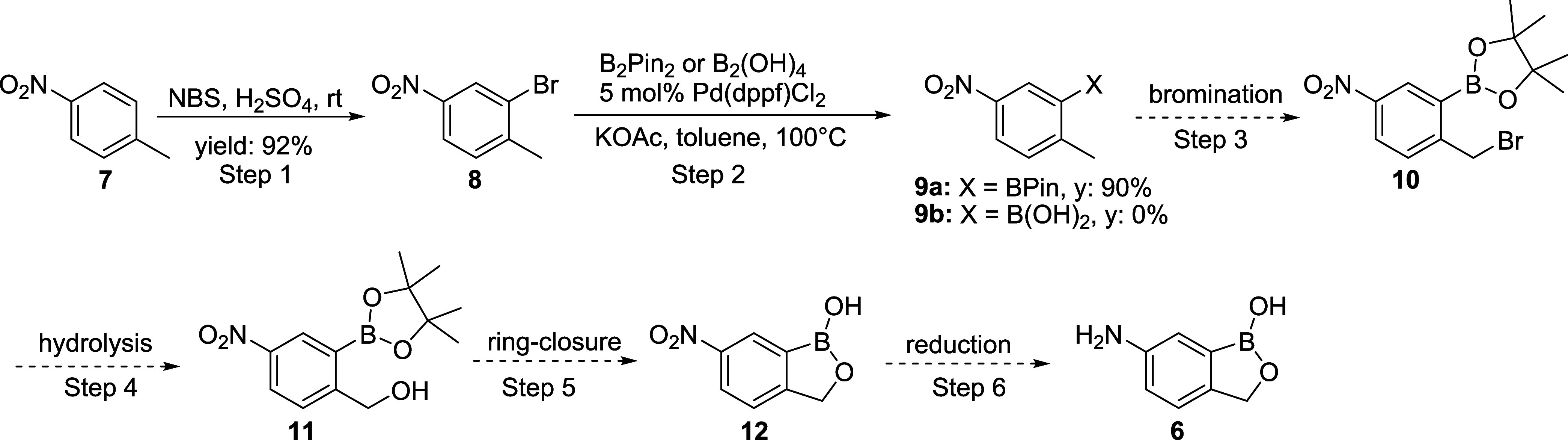
Synthesis of 6-Amino-1-hydroxy-2,1-benzoxaborolane **6** from 4-Nitrotoluene **7**

To obtain a more scalable and low-cost route for the synthesis of 6-amino-1-hydroxy-2,1-benzoxaborolane, a route utilizing 4-nitrotoluene **7** as the starting material was then investigated (Scheme 3). The proposed six-step route includes electrophilic bromination, borylation, radical bromination, hydrolysis, ring-closure, and nitroreduction and utilizes a commercially available, inexpensive starting material. We also considered routes that utilized both the pinacol boronate ester and the free boronic acid to determine whether cost or processing advantages could be realized in comparing the reactivity of these complementary functional groups.

To start this sequence, the electrophilic bromination of **7** went smoothly,^37^ affording bromoaryl compound **8** in an excellent 92% isolated yield. Initial reactions to execute the critical borylation reaction to give compound **9a** or **9b** focused on lithium-halogen exchange, followed by a reaction with a borate (iPrOBPin, B(OMe)_3_, or B(OiPr)_3_). Unfortunately, no desired product was formed under these conditions, while the starting material **8** was consumed with a messy reaction profile. Furthermore, attempts to generate and utilize a corresponding Grignard reagent failed.^38^ These failures are likely due to the incompatibility of the nitro group with the organometallic reagents or intermediates formed. Miyaura borylation proved successful, allowing advancement of **8**.^39^ Treatment of bromoaryl compound **8** with bis(pinacolato)diboron (B_2_Pin_2_) under Pd-catalysis conditions produced the borylated product **9a** in 90% yield with 5 mol % catalyst loading. Similarly, Pd-catalyzed borylation of compound **8** with tetrahydroxydiboron (B_2_(OH)_4_) gave no product **9b**. Attempts to decrease the catalyst loading in the synthesis of **9a** resulted in considerably lower conversion, challenging the use of this method as part of an overall cost-effective process. As a result, this route was abandoned in favor of the more cost-effective deaminative borylation of arylamines (see below).^40−42^

The synthesis of compound **9a** has been described in the literature from the corresponding diazonium salt of commercially available 2-methyl-5-nitroaniline (**13**, Table 2), with an excess of B_2_Pin_2_, but only on a small scale and requiring chromatographic purification.^42−44^ We focused on the optimization of this transformation for the synthesis of the intermediates **9a** or **9b**, by addressing three major issues: (1) minimize the amount of expensive diboron compound needed, thus reducing the raw material cost; (2) remove column purification to minimize the processing cost and enable scalability; and (3) diminish the process mass intensity (PMI) with a low-molecular-weight diboron source, such as B_2_(OH)_4_. As shown in Table 2, our first attempt was to utilize costly B_2_pin_2_ as the limiting reagent. Starting with the diazotization of aniline **13** (2.0 equiv) at 0 °C followed by a reaction with B_2_Pin_2_ at 25 °C, the desired product **9a** was obtained in a good isolated yield (Table 2, entry 1). Lowering the amount of aniline to 1.2 equiv (Table 2, entry 3) worked similarly well in this transformation, providing **9a** in a 56% yield. Extractive workup and subsequential trituration (with MeOH) offered a nonchromatographic purification technique to allow isolation of **9a** with >97% purity (qNMR) (Table 2, entries 1–3). Interestingly, while maintaining the borylation process at 0 °C for 3 h, the product precipitated from the reaction mixture as a yellowish solid. After a simple filtration, the desired product was obtained in a good yield (60%) with >97% purity (qNMR) (Table 2, entry 4). This process dramatically simplified the workup process. Borylation at 40 °C resulted in a low yield (Table 2, entry 5). Notably, diazotization with H_2_SO_4_ provided a cleaner HPLC profile of the reaction mixture, producing the borylated product in a higher yield (Table 2, entries 6 and 7). The optimized conditions with B_2_Pin_2_ as the boron source afforded the borylated product **9a** in a 61% isolated yield on a decagram scale (Table 2, entry 8). When tetrahydroxydiboron (B_2_(OH)_4_) was used as the boron source under the same conditions at 0 °C, no reaction was observed (Table 2, entry 9); however, increasing the reaction temperature allowed the reaction to proceed. At these higher reaction temperatures, all of the acids we screened (i.e., HCl, HBr, and H_2_SO_4_) worked similarly to afford the corresponding boronic acid **9b** in 39–45% yields (Table 2, entries 9–13); however, all of these conditions provided lower yields than reactions performed with B_2_Pin_2_. Considering that diazonium salt is the intermediate of this deaminative borylation, thermal data and runaway temperature of this transformation were investigated. DSC/TGA data of both the reaction mixture and the isolated diazonium salt indicated that the runaway temperature was greater than 90 °C and that this borylation is safe to perform under the current mild reaction conditions (see for details).

**Table 2:**
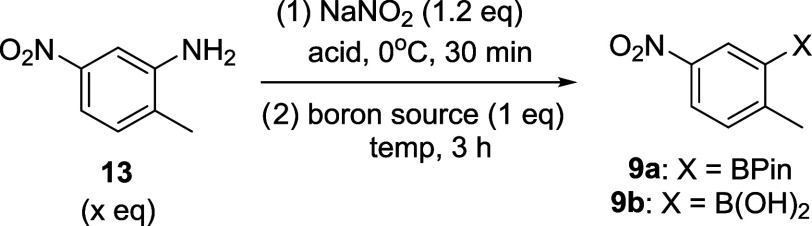
Optimization of Borylation of Aniline **13** for the Synthesis of Borylated Compounds **9a/b**^a^

entry	13 (equiv)	acid	boron source	temp (°C)	product	isolated yield (%)
1	2	HCl (6M)	B_2_Pin_2_	0–25	**9a**	60
2	1.5	HCl (6M)	B_2_Pin_2_	0–25	**9a**	61
3	1.2	HCl (6M)	B_2_Pin_2_	0–25	**9a**	56
4	1.2	HCl (6M)	B_2_Pin_2_	0	**9a**	60^b^
5	1.2	HCl (6M)	B_2_Pin_2_	0–40	**9a**	26
6	1.2	HBr (6M)	B_2_Pin_2_	0	**9a**	48
7	1.2	H_2_SO_4_ (6M)	B_2_Pin_2_	0	**9a**	77^c^
**8**^d^	**1.2**	**H**_**2**_**SO**_**4**_**(6M)**	**B**_**2**_**Pin**_**2**_	**0**	**9a**	**61**^b^
9	1.2	H_2_SO_4_ (6M)	B_2_(OH)_4_	0	**9b**	NR^e^
10	1.2	H_2_SO_4_ (6M)	B_2_(OH)_4_	0–25	**9b**	43^c^
11	1.2	HCl (6M)	B_2_(OH)_4_	0–25	**9b**	39^c^
12	1.2	HBr (6M)	B_2_(OH)_4_	0–25	**9b**	45^c^
**13**^f^,^g^	**1.2**	**H**_**2**_**SO**_**4**_**(6M)**	**B**_**2**_**(OH)**_**4**_	**0**–**25**	**9b**	**42**

a All reactions were performed with **13** (1 g, *x* equiv) and NaNO_2_ (1.2 equiv) at 0 °C in MeOH/H_2_O for 30 min, then the boron source (1 equiv) was added, and reaction time was 3 h at temperature shown in the table.

b The product was precipitated and collected by filtration.

c Assay yield based on qNMR.

d 10 g of B_2_Pin_2_ was used.

e NR: no reaction.

f 24 h.

g 10 g of B_2_(OH)_4_ was used.

With the pinacol ester **9a** and boronic acid **9b** in hand, we then studied the radical bromination of the tolyl moiety with NBS under a variety of radical initiation conditions (Table 3). With AIBN, both **9a** and **9b** reacted smoothly to yield the bromide **10a** and **10b** in 90 and 85% yields, respectively, with minor amounts of the overbrominated side products. When benzoyl peroxide (BPO) was used to initiate the bromination reaction, the pinacol boronate **9a** performed considerably better than the free boronic acid but directionally worse than AIBN in both cases, with the free boronic acid **9b** providing significant amounts of overbromination. We later found that visible incandescent light was sufficient to initiate the reaction of **9b**, providing bromide **10b** in a 80% yield.

**Table 3:**
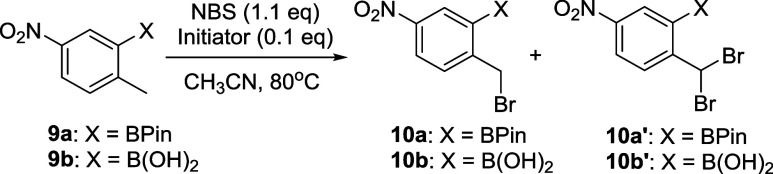
Radical Bromination of **9a/b** for the Synthesis of Bromide **10a/b**^a^

reactant	radical initiator	monobromo product (LCAP)	dibromo product (LCAP)
**9a**	AIBN	**10a**: 90%^b^	**10a**′: <5%
**9b**	AIBN	**10b**: 85%^c^	**10b**′: 15%
**9a**	BPO	**10a**: 82%^b^	**10a**′: <5%
**9b**	BPO	**10b**: 50%^c^	**10b**′: 30%
**9b**	incandescent light	**10b**: 80%^c^	**10b**′: 10%

a All reactions were performed with **9a** or **9b** (1 g, 1 equiv), NBS (1.1 equiv), initiator (0.1 equiv), or incandescent light at 80 °C in acetonitrile for 8 h.

b LCAP of the crude residue without trituration.

c LCAP of the product triturated from water.

**Scheme 4:**
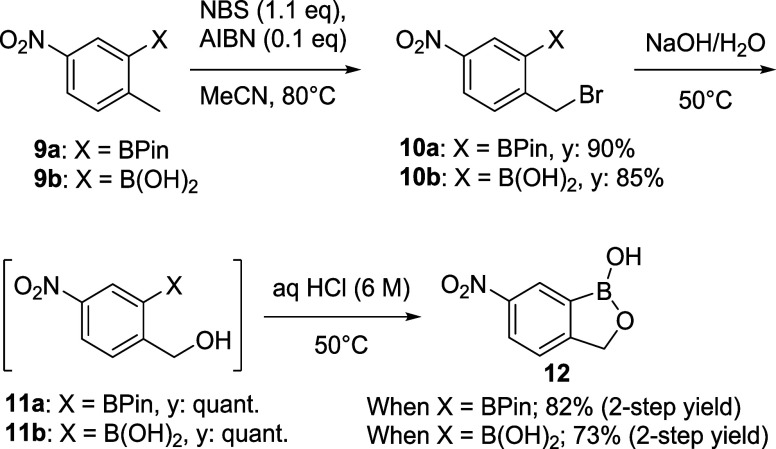
Transformation of Boronic Esters **9a** and **9b** to 6-Nitro-1-hydroxy-2,1-benzoxaborolane **12**

With access to bromides **10a** and **10b** secured, we next investigated the remaining steps to advance to the penultimate nitro-1-hydroxy-2,1-benzoxaborolane **12** with both the boronate ester and boronic acid series of compounds (Scheme 4). Given that bromides **10a** and **10b** were only semipurified by the trituration (with a purity of 90% (qNMR)), we used these materials in the following S_N_2 reaction without further purification. Thus, treatment of the semipurified products with NaOH at 50 °C yielded the benzylic alcohols **11a** and **11b** quantitatively (monitored by LCMS). Attempts to isolate the alcohols were not successful due to their instability. The resulting **11a** and **11b** benzylic alcohol mixtures were successfully telescoped to the next dehydrative ring closure reaction with aq. HCl. After removal of the solvents, the residue was triturated from EtOAc to give compound **12** with a purity >95% (qNMR). Benzoxazole **12** was obtained in a 82% overall yield in the two steps from **10a** and a 73% overall yield from **10b**.

With compound **12** in hand, we could then focus on completion of the synthesis by nitro reduction to afford the target 6-amino-1-hydroxy-2,1-benzoxaborolane **6** (Table 4). We focused our efforts on hydrogenation, but in order to ensure that the process was cost-effective, we targeted a process that could easily facilitate the recovery and/or recycling of the catalyst and that could use transfer hydrogenation instead of hydrogen gas. Initial optimization under batch conditions found that 6-amino-1-hydroxy-2,1-benzoxaborolane **6** was obtained in a 70% yield when treating compound **12** with 3 equiv of HCO_2_NH_4_ (as a hydrogen source) in the presence of 0.34 mol % Pd/C in ethanol at room temperature (rt) for 4 h. Given these encouraging results, we felt that this process could be easily adapted under flow conditions, which would offer the advantages of safety, processing costs, catalyst recyclability, etc.^45^ As shown in Table 4 (entry 1), the initial flow hydrogenation was carried out in a 1 mL Omnifit reactor, packed with 200 mg of Pd/C catalyst (5 wt %) (representing an effective catalyst loading of 0.34 mol % based on the batch scale, the volume of the packed Pd/C catalyst was 0.8 mL in the bed reactor), and an excellent isolated yield was obtained by flowing solutions of HCO_2_NH_4_ and compound **12** through the bed reactor at a flow rate of 0.1 mL/min. When the flow rate was increased to 0.4 mL/min, the same result was obtained (Table 4, entry 2). It was found that flow hydrogenation gave a shorter reaction time (1 vs 4 h) and a higher yield (93 vs 70%) than the batch condition at the same reaction scale. Lowering catalyst loading to 0.17 mol % resulted in only a 45% yield, and most of the starting material remained (Table 4, entry 3). The efficiency of this flow hydrogenation procedure was demonstrated on a 5 g scale, providing similarly good yields, giving confidence in the scalability of this process. In a 3 mL Omnifit reactor charged with 0.34 mol % of Pd/C, a solution of 5 g of compound **12** and HCO_2_NH_4_ in EtOH under a flow rate of 1.5 mL/min produced product **6** in an isolated yield of 95% within 2.5 h (Table 4, entry 4). The purification process was straightforward, and the resulting solution from the flow reactor was evaporated to dryness and triturated from ethyl acetate to afford the product with >99 wt % purity.

**Table 4:**
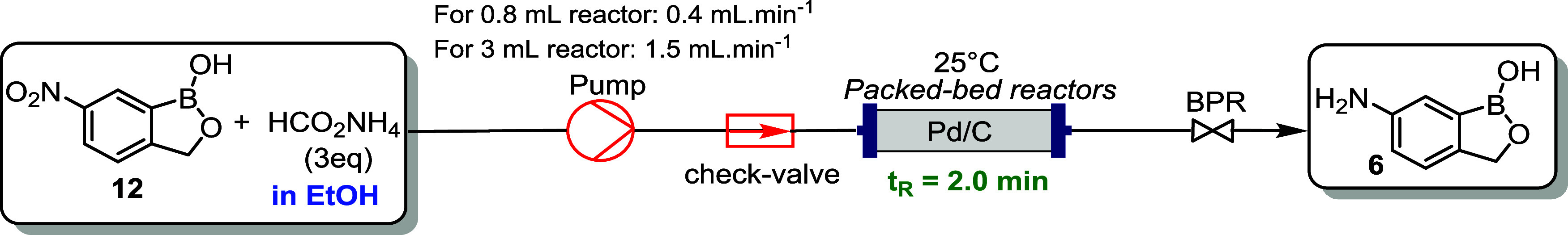
Synthesis of 6-Amino-1-hydroxy-2,1-benzoxaborolane **6** by Reduction of **12** under Continuous Flow Conditions^a^

entry	Pd/C (mol %)	time (h)	yield (%)
1^c^	0.34	4	90^b^
2^d^	0.34	1	93^b^
3^d^	0.17	1	45^e^
4^f^	0.34	2.5	95^b^

a All reactions were performed with **12** (0.5 g, 2.8 mmol, 1.0 equiv), Pd/C, and HCO_2_NH_4_ (3 equiv) in EtOH (20 mL, 40 V), at 25 °C.

b Isolated yield.

c In a 1 mL reactor, flow rate: 0.1 mL/min.

d In a 1 mL reactor, flow rate: 0.4 mL/min.

e Assay yield based on ^1^H NMR, 55% of **12** remained.

f In a 3 mL reactor, flow rate: 1.5 mL/min, 5 g of **12** was used, and **6** was obtained with >99% HPLC purity after a trituration.

**Scheme 5:**
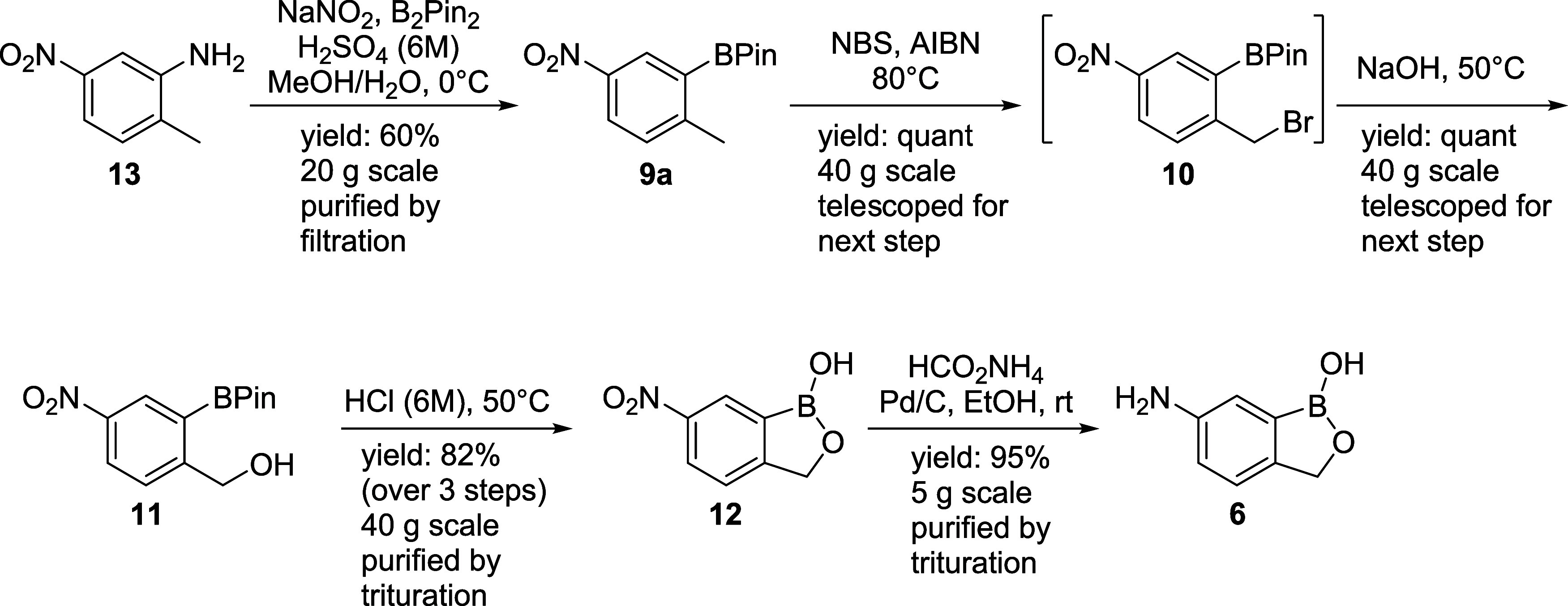
Decagram Synthesis of 6-Amino-1-hydroxy-2,1-benzoxaborolane **6** with Borylation of Amine **13**

As summarized in Scheme 5 below, through our efforts we have developed a practical five-step synthesis of **6** and have demonstrated it successfully on a decagram scale. This route gave an overall yield of 46% with >99 wt % purity without the need for column purification, which offers a variety of advantages over previously reported synthetic processes. For instance, pinacol ester **9a** gave cyclized product **12** in three steps with an overall yield of 82%. Also, the final transhydrogenation of the nitro-compound **12** under continuous flow conditions afforded the 6-amino-1-hydroxy-2,1-benzoxaborolane **6** in excellent yield. This route provides an effective protocol for the synthesis of 6-amino-1-hydroxy-2,1-benzoxaborolane **6**.

## Conclusions

In conclusion, two new approaches for the synthesis of 6-amino-1-hydroxy-2,1-benzoxaborolane (**6**) from inexpensive and readily available raw materials have been developed. The sequence based on 4-tolunitrile utilized a Hofmann rearrangement as the key transformation with a 40% overall yield. The more promising and practical second approach employed 2-methyl-5-nitroaniline as the starting material. This strategy featured the borylation of aniline, to provide either pinacol boronate ester or free boronic acid, and concluded with a continuous flow nitro reduction, offering up to 46% overall yield with a facile isolation process for intermediates and 6-amino-1-hydroxy-2,1-benzoxaborolane. Both strategies have been demonstrated on a multigram scale, bypassing the challenging nitration step and better leveraging the commercially available, inexpensive starting materials. This proof-of-concept work advances the synthesis of 6-amino-1-hydroxy-2,1-benzoxaborolane and may be useful in further efforts to optimize the process toward affordable commercial manufacture of DNDI-6148 (and similar benzoxaborole drugs). With the studies showing that DNDI-6148 could potentially be developed for treatment of cutaneous leishmaniasis (CL) and Chagas disease in addition to VL, this work could have a significant impact on enabling access of DNDI-6148 to those in dire need.

## Experimental Section

### General Information

Reagents and solvents were obtained from commercial suppliers and used as received, unless otherwise indicated. Reactions were carried out in oven-dried (120 °C) glassware that was assembled while hot and cooled to ambient temperature under an inert atmosphere. All reactions were carried out under inert atmosphere (N_2_) unless otherwise noted. Reactions were monitored by TLC (precoated silica gel 60 F254 plates, EMD Chemicals), HPLC, or LC/MS using various methods. TLC was visualized with UV light or by treatment with phosphomolybdic acid (PMA), ninhydrin, and/or KMnO_4_. Flash chromatography was performed on a Teledyne ISCO Combi-Flash NEXTGEN 300+ and/or a Biotage Isolera using solvents as indicated. HRMS was recorded using a PerkinElmer Axion 2 ToF MS, ionization mode: positive with scan range: 100–1000 *m*/*z*, flight tube voltage: 8 kV, spray voltage: 3.5 kV, solvent: methanol. The ^1^HNMR and ^13^CNMR spectra were routinely recorded on a Bruker Avance III HD Ascend 600 MHz spectrometer. The NMR solvents used were CDCl_3_, CD_3_OD, or DMSO-*d*_6_ as indicated. Tetramethylsilane (TMS) was used as an internal standard. Coupling constants *J* are reported in hertz (Hz). The following abbreviations were used to designate signal multiplicity: s, singlet; d, doublet; t, triplet; q, quartet, p, pentet; dd, doublet of doublets; ddd, doublet of doublet of doublets; dt, double of triplets; ddt, doublet of doublet of triplets; m, multiplet; br, broad. 1,3,5-trimethoxybenzene and/or triphenylmethane were used as internal standards for quantitative ^1^H NMR.

#### Synthesis of 3-Bromo-4-methylbenzonitrile (**2**) from *p*-Tolunitrile (**1**)

To a 500 mL round-bottom flask equipped with a magnetic stir bar was added 4-tolunitrile **1** (5.0 g, 1.0 equiv, 42.7 mmol) and 100 mL of aqueous sulfuric acid (50:50 ratio by volume of conc. H_2_SO_4_ to water). The flask was wrapped with aluminum foil to prevent competitive free-radical reactions. The mixture was stirred for 10 min, whereupon *N*-bromosuccinimide (8.4 g, 1.1 equiv, 46.9 mmol) was added to the flask slowly for 5 min via a solid addition funnel. The mixture was then stirred at 25 °C for 12 h and then analyzed via GC/MS. After completion, the reaction mixture was extracted with DCM (100 mL × 3). The combined organics were washed with brine (100 mL), dried over sodium sulfate, filtered, and concentrated *in vacuo* to afford 8.54 g of crude material. This material was passed through a SiO_2_ plug and washed thrice with 5% EtOAc in hexanes (100 mL each) to give 7.78 g of 3-bromo-4-methylbenzonitrile **2** as a white solid (39.7 mmol, 92.9%) with 99% purity via qNMR.

^1^H NMR (600 MHz, DMSO-*d*_6_): δ = 8.11 (d, *J* = 1.5 Hz, 1H), 7.76 (dd, *J* = 1.7, 7.9 Hz, 1H), 7.55 (d, *J* = 7.9 Hz, 1H), 2.41 (s, 3H). ^13^C NMR (150 MHz, DMSO-*d*_6_): δ 143.8, 135.2, 131.8, 131.4, 124.5, 117.5, 110.5, 22.8. MS (*m*/*z*) (M + H): calcd for C_8_H_7_BrN 195, found 195. Melting point 43–45 °C.

#### Synthesis of 3-Bromo-4-(hydroxymethyl)benzonitrile (**3**) from 3-Bromo-4-methylbenzonitrile (**2**)

To a 500 mL three-neck round-bottom flask equipped with a magnetic stir bar was added 3-bromo-4-methylbenzonitrile (10.0 g, 1.0 equiv, 51.0 mmol) and acetonitrile (150 mL). *N*-Bromosuccinimide (13.6 g, 1.5 equiv, 76.5 mmol) was then added to the mixture and stirred at 25 °C for 12 h in the presence of a light source (see the picture below). The reaction mixture was then analyzed via GC/MS, and upon confirming consumption of **2**, the reaction was concentrated *in vacuo*. The crude material was partitioned between DCM (100 mL) and DI H_2_O (100 mL), and the aqueous layer was extracted twice with DCM (100 mL each). The organic layers were combined, washed with DI H_2_O (100 ml) and brine (100 ml), and dried over Na_2_SO_4_. The material was then filtered and concentrated *in vacuo* to give crude 3-bromo-4-(bromomethyl)benzonitrile (14.63 g) as a yellow solid (confirmed by ^1^HNMR). To the crude material was added 1,4-dioxane (80 mL), water (120 mL), and calcium carbonate (23.5 g, 4.6 equiv, 234.6 mmol). This mixture was heated at 100 °C for 16 h and then analyzed via LC/MS for the starting material consumption. Upon confirmation, the mixture was cooled to room temperature and filtered through celite. The filtrate was partitioned between water (100 mL) and EtOAc (100 mL). The aqueous layer was extracted twice with EtOAc (100 mL each). The combined organics were washed with water (100 mL) and brine (100 mL), dried over anhydrous Na_2_SO_4_, filtered, and concentrated *in vacuo* to give 12.22 g of crude as a tan solid. This resulting solid was recrystallized with 100 mL of DCM:MeOH (80:10, v/v) to obtain 8.97 g of 3-bromo-4-(hydroxymethyl)benzonitrile **3** as a white powder (42.3 mmol, 82.9%) with 94% purity via weight % HPLC analysis.

^1^H NMR (600 MHz, DMSO-*d*_6_): δ 8.12 (d, *J* = 1.5 Hz, 1H), 7.88 (dd, *J* = 1.4, 8.0 Hz, 1H), 7.70 (d, *J* = 8.1 Hz, 1H), 5.71 (s, 1H), 4.55 (br. s., 2H). ^13^C NMR (150 MHz, DMSO-*d*_6_): δ 147.2, 135.1, 131.6, 128.3, 121.0, 117.6, 111.1, 62.5. MS (*m*/*z*) (M + H): calcd for C_8_H_7_BrNO 212, found 212. Melting point 135–137 °C.

#### Synthesis of 1-Hydroxy-1,3-dihydrobenzo[*c*][1,2]oxaborole-6-carbonitrile (**4**) from 3-Bromo-4-(hydroxymethyl)benzonitrile (**3**)

A 1000 mL three-necked round-bottom flask was charged with a stir bar, and then 3-bromo-4-(hydroxymethyl)benzonitrile **3** (10.0 g, 1 equiv, 47.2 mmol) and tetrahydrofuran (300 mL, Sigma-Aldrich, anhydrous) were added under N_2_. The reaction vessel was cooled at −77 °C, and then triisopropyl borate (17.7 g, 21.8 mL, 2 equiv, 94.3 mmol) was added. The mixture was stirred for 20 min before the addition of 2.5 M *n*-butyllithium in hexanes (7.55 g, 47.2 mL, 2.50 M, 2.5 equiv, 117.9 mmol) dropwise in three separate portions at −77 °C. The mixture was removed from the cooling bath and allowed to warm to room temperature and stirred for 16 h under an N_2_ atmosphere. After this, the mixture was quenched with 1 M HCl (100 mL) and extracted thrice with ethyl acetate (100 mL each). The combined organic layer was washed with brine (100 mL), dried over anhydrous Na_2_SO_4_, and then concentrated *in vacuo* to afford 12.9 g of a yellow solid. This solid was triturated with 100 mL of a suitable solvent (i.e., Et_2_O, MTBE, DCM, and/or hexanes) to give 6.67 g of 1-hydroxy-1,3-dihydrobenzo[*c*][1,2]oxaborole-6-carbonitrile **4** as a pale-yellow solid (42.0 mmol, 89% yield) with 96% purity via qNMR.

^1^H NMR (600 MHz, DMSO-*d*_6_): δ 9.51 (s, 1H), 8.10 (s, 1H), 7.91 (dd, *J* = 1.7, 7.9 Hz, 1H), 7.64 (dd, *J* = 0.6, 7.9 Hz, 1H), 5.08 (s, 2H). ^13^C NMR (150 MHz, DMSO-*d*_6_): δ 158.7, 134.6, 133.9, 122.9, 119.2, 110.0, 70.2. MS (*m*/*z*) [M + H]^+^: calcd for C_8_H_7_BNO_2_ 160, found 160. Melting Point 197–201 °C.

#### Synthesis of 1-Hydroxy-1,3-dihydrobenzo[*c*][1,2]oxaborole-6-carboxamide (**5**) from 1-Hydroxy-1,3-dihydrobenzo[*c*][1,2]oxaborole-6-carbonitrile (**4**)

To a 500 mL round-bottom flask, charged with a stir bar, were added 1-hydroxy-1,3-dihydrobenzo[*c*][1,2]oxaborole-6-carbonitrile 4 (6.65 g, 1 equiv, 41.8 mmol) and methanesulfonic acid (120.6 g, 81.4 mL, 30 equiv, 1.26 mol). The reaction mixture was heated at 90 °C for 16 h under N_2_ atmosphere. After this, the reaction was analyzed via LC/MS to confirm the consumption of the starting material. The reaction mixture was then neutralized (pH 6–7) with 6 M NaOH (30 mL), concentrated *in vacuo* onto C18 silica gel, and purified via reverse-phase chromatography with 5% ACN in H_2_O plus 0.1% formic acid to give 5.48 g of 1-hydroxy-1,3-dihydrobenzo[*c*][1,2]oxaborole-6-carboxamide **5** as a white solid (31.0 mmol, 74.0% yield) with a 96% purity via qNMR. This corresponds to a corrected yield of 71.0%.

^1^H NMR (600 MHz, DMSO-*d*_6_): δ 9.30 (br. s., 1H), 8.24 (d, *J* = 0.73 Hz, 1H), 7.98 (br. s., 1H), 7.95 (dd, *J* = 1.65, 7.89 Hz, 1H), 7.46 (dd, *J* = 0.55, 7.89 Hz, 1H), 7.33 (br. s., 1H), 5.03 (s, 2H). ^13^C NMR (150 MHz, DMSO-*d*_6_): δ 168.3, 156.8, 133.3, 130.0, 129.8, 121.1, 69.9. MS (*m*/*z*) [M + H]^+^: calcd for C_8_H_9_BNO_3_ 178, found 178. Melting Point 209–211 °C.

#### Synthesis of 6-Aminobenzo[*c*][1,2]oxaborol-1(3*H*)-ol (**6**) from 1-Hydroxy-1,3-dihydrobenzo[*c*][1,2]oxaborole-6-carboxamide (**5**)

To a solution of 1-hydroxy-1,3-dihydrobenzo[*c*][1,2]oxaborole-6-carboxamide **5** (2.00 g, 1 equiv, 11.3 mmol) and sodium hydroxide (ultradry, 2.49 g, 5.5 equiv, 62.2 mmol) in H_2_O (50 mL) was added trichloroisocyanuric acid (880 mg, 0.335 equiv, 3.79 mmol) at 0 °C. This solution was stirred for 2 h, then allowed to warm to 25 °C, and stirred for an additional 12 h. The reaction was then neutralized (pH 6–7) with 1 M HCl (40 mL) and extracted three times with EtOAc (100 mL each). The organics were washed with brine (50 mL), dried over Na_2_SO_4_, and concentrated *in vacuo* to afford a yellow solid. This solid was recrystallized from MTBE (20 mL) to afford 1.41 g of 6-aminobenzo[*c*][1,2]oxaborol-1(3*H*)-ol **6** (9.47 mmol, 83.7%) as a light-yellow solid with 96% purity via weight % HPLC analysis. This corresponds to a corrected yield of 80.4%.

^1^H NMR (600 MHz, DMSO-*d*_6_): δ 8.91 (s, 1H), 7.03 (d, *J* = 8.07 Hz, 1H), 6.89 (d, *J* = 2.02 Hz, 1H), 6.70 (dd, *J* = 2.20, 8.07 Hz, 1H), 4.98 (br. S., 2H), 4.81 (s, 2H). ^13^C NMR (150 MHz, DMSO-*d*_6_): δ 147.5, 141.4, 121.4, 117.6, 114.6, 69.6. HRMS (ESI) *m*/*z*: [M + H]^+^ calcd for C_7_H_9_BNO_2_ 150.0648, found 150.0625. Melting Point 147–150 °C.

#### Synthesis of 4,4,5,5-Tetramethyl-2-(2-methyl-5-nitrophenyl)-1,3,2-dioxaborolane (**9a**) via Diazotization

To an ice-cold suspension of 2-methyl-5-nitroaniline **13** (7.19 g, 1.2 equiv, 47.2 mmol) and MeOH (60 mL) was added H_2_SO_4_ (59.0 mL, 6.0 molar, 9.3 equiv, 354.1 mmol). The internal temperature was monitored by J-Kem. The resulting mixture was cooled to 0 °C, and a solution of sodium nitrite (4.1 g, 1.5 equiv, 59.0 mmol) in water (60 mL) was added dropwise using an addition funnel, while maintaining internal temperature below 10 °C. The mixture was stirred at 0 °C for 30 min, and at this time the suspension became a clear solution. After consumption of the starting material aniline (monitored by TLC and HPLC), a solution of 4,4,4′,4′,5,5,5′,5′-octamethyl-2,2′-bi(1,3,2-dioxaborolane) (10 g, 1.0 equiv, 39.4 mmol) in methanol (60 mL) was added dropwise at 0 °C. The resulting mixture was stirred for 3 h at rt, and the precipitated yellowish solid was collected by filtration to give a pure product **9a** (6.3 g, 61%). The filtrate contained mainly the deamination side product **SP1** (1.3 g, 24%).

^1^H NMR (600 MHz, CDCl_3_) δ/ppm: 8.59 (d, *J* = 2.6 Hz, 1H), 8.12 (dd, *J* = 8.4, 2.6 Hz, 1H), 7.29 (d, *J* = 8.4 Hz, 1H), 2.62 (s, 3H), 1.35 (s, 12H). ^13^C NMR (150 MHz, CDCl_3_) δ/ppm: 152.9, 145.8, 130.8, 125.5, 84.4, 25.0, 22.6. MS (*m*/*z*) (M + H): calcd for C_13_H_19_BNO_4_ 264, found 264. Melting Point 89–92 °C.

##### 1-Methyl-4-nitrobenzene (SP1)

^1^H NMR (600 MHz, CDCl_3_) δ/ppm: 8.08 (d, *J* = 8.6 Hz, 1H), 7.29 (dd, *J* = 8.6 Hz, 1H), 2.44 (s, 3H). ^13^C NMR (150 MHz, CDCl_3_) δ/ppm: 146.1, 129.9, 133.6, 21.7.

#### Synthesis of (2-Methyl-5-nitrophenyl)boronic Acid (**9b**)

To an ice-cooled solution of 2-methyl-5-nitroaniline **13** (40.73 g, 1.2 equiv, 267.7 mmol) in MeOH (200 mL) was added H_2_SO_4_ (345.8 mL, 6.0 M, 9.3 equiv, 2.0 mol) slowly to control the internal temperature within 20 °C. The resulting suspension was cooled to 0 °C, and sodium nitrite (23.1 g, 1.5 equiv, 334.6 mmol) in water (60.0 mL) was added dropwise using an addition funnel, the care being taken not to raise the internal temperature above 10 °C. The resulting mixture was stirred at 0 °C for 30 min. During the course, a clear solution was formed. After consumption of aniline and formation of the diazonium (checked by TLC and HPLC), tetrahydroxy diborane (20.0 g, 1.0 equiv, 223.1 mmol) was added portion wise as a solid and the resulting mixture was stirred at rt for 24 h. After completion (checked by HPLC), methanol was removed under vacuum, and the remaining aqueous solution was extracted with EtOAc (200 mL × 3). The combined organic layer was dried over Na_2_SO_4_ and rotavaped to dryness to give the crude product as a mixture of **14** and **SP1**. The crude mixture was washed with hexane (50 mL × 3) to remove the side product **SP1** (5.9 g, 20% yield), and the resulting brown solid was triturated from EtOAc (50 mL) to give the pure **9b** as pale-yellow solid (18.0 g, 45% yield with 98% qNMR purity).

^1^H NMR (600 MHz, DMSO-*d*_6_) δ/ppm: 8.27 (d, *J* = 2.6 Hz, 1H), 8.08 (dd, *J* = 8.5, 2.6 Hz, 1H), 7.40 (d, *J* = 8.4 Hz, 1H), 2.52 (s, 3H). ^13^C NMR (150 MHz, DMSO-*d*_6_) δ/ppm: 152.0, 144.9, 130.6, 127.7, 123.5, 22.2. MS (*m*/*z*) (M + H): calcd for C_7_H_9_BNO_4_ 182, found 182. Melting Point 56–57 °C.

#### Synthesis of 2-(2-(Bromomethyl)-5-nitrophenyl)-4,4,5,5-tetramethyl-1,3,2-dioxaborolane (**10a**) via Radical Bromination of (**9a**)

A mixture of 4,4,5,5-tetramethyl-2-(2-methyl-5-nitrophenyl)-1,3,2-dioxaborolane **9a** (41 g, 1.0 equiv, 156 mmol), BPO (3.77 g, 0.1 equiv, 15.6 mmol), and NBS (41.60 g, 233.75 mmol, 1.5 equiv) in MeCN (500 mL) was stirred at 80 °C for 8 h. After HPLC and LCMS showed that the starting material was consumed completely, the reaction mixture was cooled to 25 °C and concentrated under reduced pressure to give a crude residue. The residue was quenched by saturated Na_2_SO_3_ (500 mL) and extracted with EtOAc (300 mL × 3). The combined organic layer was washed with brine (200 mL), dried over Na_2_SO_4_, and concentrated under reduced pressure to give the crude product **10a** in a quantitative yield. The crude mixture was used for the next step without further purification.

#### Synthesis of (2-(Bromomethyl)-5-nitrophenyl)boronic Acid (**10b**) via Radical Bromination of (**9b**)

A mixture of (2-methyl-5-nitrophenyl)boronic acid **9b** (10.0 g, 1.0 equiv, 55.3 mmol), AIBN (1.1 g, 0.1 equiv, 5.52 mmol), and NBS (10.8 g, 1.1 equiv, 60.8 mmol) in MeCN (200 mL) was stirred at 25 °C for 10 min. The resulting clear solution was then stirred at 80 °C for 1 h. After completion (monitored by HPLC), the reaction mixture was cooled to 25 °C and concentrated to dryness under reduced pressure. The residue was then triturated with H_2_O (30 mL × 2) to give a crude solid product (11.9 g, 83% yield) as a mixture of **10b** 80% monobromo and **10b**′ 10% dibromo. The crude mixture was used for the next step without further purification. For analytical data, the mixture was purified by prep-HPLC.

##### (2-(Bromomethyl)-5-nitrophenyl)boronic Acid (**10b**)

^1^H NMR (600 MHz, DMSO-*d*_6_): δ 8.36 (d, *J* = 2.51 Hz, 1H), 8.18 (dd, *J* = 8.5, 2.5 Hz, 1H), 7.67 (d, *J* = 8.5 Hz, 1H), 4.99 (s, 2H), 3.54 (brs, 2H). ^13^C NMR (150 MHz, DMSO-*d*_6_): δ 160.7, 147.3, 132.5, 125.9, 125.8, 123.3, 70.3. LCMS (ESI) *m*/*z*: [M + H]^+^ calcd for C_7_H_8_BBrNO_4_ 259, found 259.

##### (2-(Dibromomethyl)-5-nitrophenyl)boronic Acid (**10b′**)

^1^H NMR (600 MHz, DMSO-*d*_6_): δ 8.38 (d, *J* = 2.5 Hz, 1H), 8.32 (dd, *J* = 8.5, 2.5 Hz, 1H), 8.14 (d, *J* = 8.5 Hz, 1H), 7.84 (s, 1H). ^13^C NMR (150 MHz, DMSO-*d*_6_): δ 152.9, 147.3, 132.6, 131.5, 129.1, 125.9, 40.4. LCMS (ESI) *m*/*z*: [M + H]^+^ calcd for C_7_H_7_BBrNO_4_ 337, found 337.

#### Synthesis of 6-Nitrobenzo[*c*][1,2]oxaborol-1(3*H*)-ol (**12**) from **10a**

To a solution of 2-(2-(bromomethyl)-5-nitrophenyl)-4,4,5,5-tetramethyl-1,3,2-dioxaborolane **10a** (50 g, 146.20 mmol, 1.0 equiv) in THF (500 mL) and H_2_O (100 mL) was added NaOH (17.54 g, 438.61 mmol, 3 equiv), and the reaction mixture was stirred at 50 °C for 2 h. Once completed (monitored by TLC and LCMS), the mixture was cooled down to 25 °C. To this was added HCl (6 M, 219.30 mL, 9 equiv) at 25 °C, and the reaction mixture was stirred at 50 °C for an additional 8 h. TLC showed that the starting material was consumed completely. The reaction mixture was cooled to rt and extracted with EtOAc (200 mL × 3). The combined organic layer was washed with brine (100 mL), dried over Na_2_SO_4_, and concentrated under reduced pressure to give a crude residue. The residue was triturated from EtOAc (50 mL) to give desired compound **12** (21 g, 82%) as a yellow solid.

^1^H NMR (600 MHz, DMSO-*d*_6_) δ/ppm: 8.52–8.45 (m, 1H), 8.27–8.19 (m, 1H), 7.65–7.58 (m, 1H), 5.07 (s, 2H). ^13^C NMR (150 MHz, DMSO-*d*_6_) δ/ppm: 160.6, 147.1, 132.3 (C–B), 125.6, 125.5, 122.9, 70.1. MS (*m*/*z*) [M + H]^+^: calcd for C_7_H_7_BNO_4_ 180, found 180. Melting Point 175–177 °C.

#### Synthesis of 6-Nitrobenzo[*c*][1,2]oxaborol-1(3*H*)-ol (**12**) from **10b**

To a solution of the mixture of (2-(bromomethyl)-5-nitrophenyl)boronic acid **10b** and **10b**′ (10.0 g, 1.0 equiv, 38.5 mmol) in THF (170 mL) and H_2_O (30 mL) was added sodium hydroxide (4.6 g, 3.0 equiv, 115.4 mmol). The reaction mixture was stirred at 50 °C for 2 h; during this time, a lot of solid was precipitated out. After the reaction was complete (checked by LCMS and HPLC), the mixture was cooled to 25 °C, and then aq HCl (32 mL, 6 molar, 5.0 equiv, 192.4 mmol) was added; at this time, the reaction mixture became completely homogeneous. The resulting mixture was stirred at 50 °C for 8 h. After the starting material was consumed completely (monitored by TLC), the reaction mixture was cooled to rt and concentrated under reduced pressure to about a 50 mL volume. The resulting suspension was filtered, and the filter cake was rinsed with ∼30 mL of water and then dried under a high vacuum to give a crude solid which on titration with EtOAc (12 mL) gave a pure yellow solid **12** (5.23 g, 72% yield, 95% qNMR purity). The analytical data matched well with a previously prepared compound.

### Procedure for the Synthesis of 6-Aminobenzo[*c*][1,2]oxaborol-1(3*H*)-ol (**6**) in a Continuous Flow Reactor

A premixed solution of 6-nitrobenzo[*c*][1,2]oxaborol-1(3*H*)-ol **12** (5.0 g, 1.0 equiv, 28.0 mmol) and ammonium formate (7.0 g, 4.0 equiv, 111 mmol) in EtOH (200 mL) was pumped by using a vaportec-E series pump to the prepacked Pd/C (2.0 g, 0.34 mol %) Omnifit-reactor (3 mL) at 1.5 mL/min flow rate and the reaction mixture was collected at the end port. The reaction completed in 2 h to give the crude product as a brown solid (5.2 g). The crude product was further purified using trituration with EtOAc (5 mL) to afford the pure product as a yellow solid **6** (3.96 g, 92% yield, 97% qNMR purity). The analytical data of the product matched very well with the previously prepared compound.

## Supporting Information Available

Detailed HPLC/LCMS method development, copies of NMR spectra of all compounds, synthesis, and DSC and TGA studies of diazonium salt (PDF)
