# Supplementary material for: Practical Synthesis of 6-Amino-1-hydroxy-2,1-benzoxaborolane: A Key Intermediate of DNDI-6148
Source: Org Process Res Dev. 2024 Mar 29;28(4):1213–23. doi: 10.1021/acs.oprd.4c00031 (PMC11036395; doi:10.1021/acs.oprd.4c00031)
Supplement: Supplementary file 1 — op4c00031_si_001.pdf [file op4c00031_si_001.pdf]

Practical synthesis of 6-amino-1-hydroxy-2,1-benzoxaborolane: a key intermediate of DNDI-6148

Pankaj V. Khairnar[1Δ], *John M. Saathoff*[1Δ], *Daniel W. Cook*[1], *Samuel R. Hochstetler*[1], *Urvish Pandya*[2], *Stephen J. Robinson*[2], *Vijay Satam*[2], *Kai O. Donsbach*[1], *B. Frank Gupton*[1], *Li-Mei Jin*[1]*, *Charles S. Shanahan*[1]*

[1]Medicines for All Institute, Virginia Commonwealth University, Richmond, VA, 23284-3068.

[2]Drugs for Neglected Diseases initiative, 15 Chemin Camille-Vidart, 1202 Geneva, Switzerland.

[Δ]These authors contributed equally.

*Corresponding author. Email: jinl3@vcu.edu; shanahanc@vcu.edu

GENERAL METHODS

Reagents and solvents were obtained from commercial suppliers and used as received unless otherwise indicated. Reactions were carried out in oven-dried (120 °C) glassware, that was assembled while hot, and cooled to ambient temperature under an inert atmosphere. All reactions were carried out under inert atmosphere (N_2) unless otherwise noted. Reactions were monitored by TLC (precoated silica gel 60 F254 plates, EMD Chemicals), HPLC or LC/MS using various methods. TLC was visualized with UV light or by treatment with Phosphomolybdic acid (PMA), ninhydrin, and/or $KMnO_4$. Flash chromatography was performed on a Teledyne ISCO Combi-Flash NEXTGEN 300+ and/or a Biotage Isolera using solvents as indicated. HRMS was recorded using Perkin Elmer Axion 2 ToF MS, ionization mode: positive with scan range: 100 - 1000 m/z, flight tube voltage: 8 kV, spray voltage: 3.5 kV, solvent: methanol.^{1}HNMR and ^{13}CNMR spectra were routinely recorded on Bruker Avance III HD Ascend 600 MHz spectrometer. The NMR solvents used were $CDCl_3$, CD_3OD or DMSO-d_6 as indicated. Tetramethylsilane (TMS) was used as an internal standard. Coupling constants J are reported in hertz (Hz). The following abbreviations were used to designate signal multiplicity: s, singlet; d, doublet; t, triplet; q, quartet, p, pentet; dd, doublet of doublets; ddd, doublet of doublet of doublets; dt, double of triplets; ddt, doublet of doublet of triplets; m, multiplet; br, broad. 1,3,5-trimethoxybenzene and/or triphenylmethane, were used as internal standards for quantitative ^{1}H-NMR.

HPLC/LCMS Method

LC with UV detection was used for the analysis of reaction mixtures and isolated product and intermediates. An Agilent 1100 series LC equipped with a diode array detector was used with the parameters shown below.

**Column**	Phenomenex Kinetex Phenyl Hexyl (150 x 4.6 mm; 5 µm)
**Flow Rate**	2.0 mL/min
**Column Temperature**	40 °C
**Injection Volume**	1 µL
**Mobile Phase A**	0.1% H_3PO_4 in water*
**Mobile Phase B**	Methanol

Gradient Table	Time (min)	%A	%B
	0.0	80%	20%
	9.0	25%	75%
	12.0	25%	75%
**Post-Run Equilibration**	2 min		
**Detection Wavelength**	210 nm		

Table S1. Synthesis of amide 5 by hydrolysis of nitrile 4.

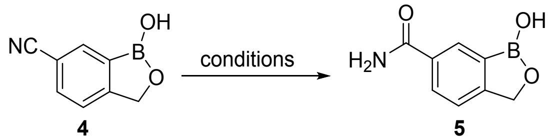

Entry	Conditions	Isolated yield of 5
1 (**4**: 1 g)	NaOH, iPrOH, 90 ˚C, 16h	~18 A%
2 (**4**: 1 g)	LiOH, iPrOH, 90 ˚C, 16h	NR
3 (**4**: 1 g)	KOH, iPrOH, 90 ˚C, 16h	~60 A% (failed to isolate)
4 (**4**: 1 g)	H_2SO_4, 90 ˚C, 1h	49%
5 (**4**: 10 g)	H_2SO_4, 90 ˚C, 1h	53%
6 (**4**: 1 g)	MsOH, 90 ˚C, 16h	72%
7 (**4**: 10 g)	MsOH, 90 ˚C, 16h	78%
8 (**4**: 1 g)	TFA, 90 ˚C, 16h	31%
9 (**4**: 1 g)	TfOH, 90 ˚C, 16h	5%[a]
10 (**4**: 1 g)	H_2SO_4, 90 ˚C, 16h	0[a]
11 (**4**: 1 g)	HCl, 90 ˚C, 16h	12[b]
11 (**4**: 1 g)	$NaHSO_4$, 90 ˚C, 16h	NR
11 (**4**: 1 g)	$NaHSO_3$, 90 ˚C, 16h	NR

[a]SM was consumed. Decomposition occurred. [b]76 A% carboxylic acid was formed.

Table S2. Condition screening for the borylation of 2-bromo-1-methyl-4-nitrobenzene (8).

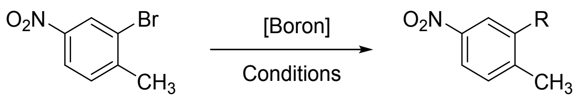

8 **9a:** R = BPin
9b: R = $B(OH)_2$

Entry[a]	[Boron]	Conditions	Solvent	Temp /°C	A%[b]
1	$B(OMe)_3$	iPrMgCl-LiCl	THF	-78	ND
2	$B(OiPr)_3$	iPrMgCl-LiCl	THF	-78	ND
3	$B(OiPr)_3$	n-BuLi	THF	-78	ND
4	iPrO-BPin	n-BuLi	THF	-78	ND
5	BH_3-NH(iPr)$_2$	Mg, PhLi	THF	70	ND
6	$B(OiPr)_3$	Mg	THF	-78	NR
7	B_2Pin_2	5 mol% Pd(PPh_3)Cl_2, PPh_3, KOAc	1,4-Dioxane	100	20
8	B_2Pin_2	5 mol% Pd(OAc)$_2$, PPh_3, KOAc	1,4-Dioxane	100	ND
9	B_2Pin_2	5 mol% Pd(OAc)$_2$, Xphos,, KOAc	EtOH	80	10
10	B_2Pin_2	5 mol% Pd(dppf)Cl_2, KOAc	1,4-Dioxane	100	>90
11	B_2Pin_2	1% Pd(dppf)Cl2, KOAc	1,4-Dioxane	100	~30
12	B_2Pin_2	1% Pd(OAc)2, dppf, KOAc	1,4-Dioxane	100	~56
13	B_2Pin_2	0.1% Pd(dppf)Cl2, KOAc	1,4-Dioxane	100	~7
14	B_2Pin_2	10 mol% Pd(OAc)2	1,4-Dioxane	100	ND
15	B_2Pin_2	10 mol% PdCl2	1,4-Dioxane	100	ND
16	B_2Pin_2	10 mol% Pd/C	1,4-Dioxane	100	ND
17	$B_2(OH)_4$	5 mol% Pd(PPh3)Cl_2, PPh_3, KOAc	EtOH	80	ND
18	$B_2(OH)_4$	5 mol/% Pd(OAc)2, Xphos,, KOAc	EtOH	80	20
19	$B_2(OH)_4$	5 mol% Pd(dppf)Cl2, KOAc	EtOH	80	ND
20	$B_2(OH)_4$	5 mol% Xphos-Pd-G2, KOAc	EtOH	80	ND

[a]Reaction conditions: For metal-halo exchange borylation: organometal (1.5 eq) and/or Mg (2 eq), 2-bromo-1-methyl-4-nitrobenzene (1.0 eq.), [Boron] (2 eq.), Solvent (10V); For Pd-borylation: Pd-Catalyst (X mol%), 2-bromo-1-methyl-4-nitrobenzene (1.0 eq.), [Boron] (1.1 eq.), potassium acetate (2.0 eq.), Solvent (10V). [b]Yields were calculated using HPLC A% at 210 nm.

Setup for synthesis of 3-bromo-4-(hydroxymethyl)benzonitrile (3) from 3-bromo-4-methylbenzonitrile (2)

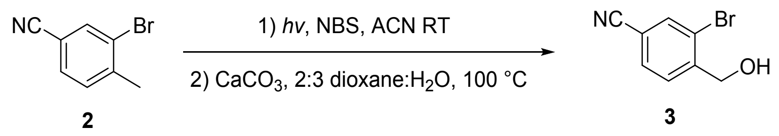

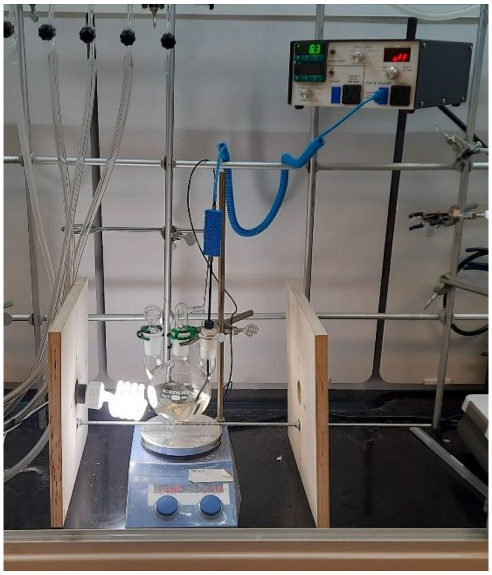

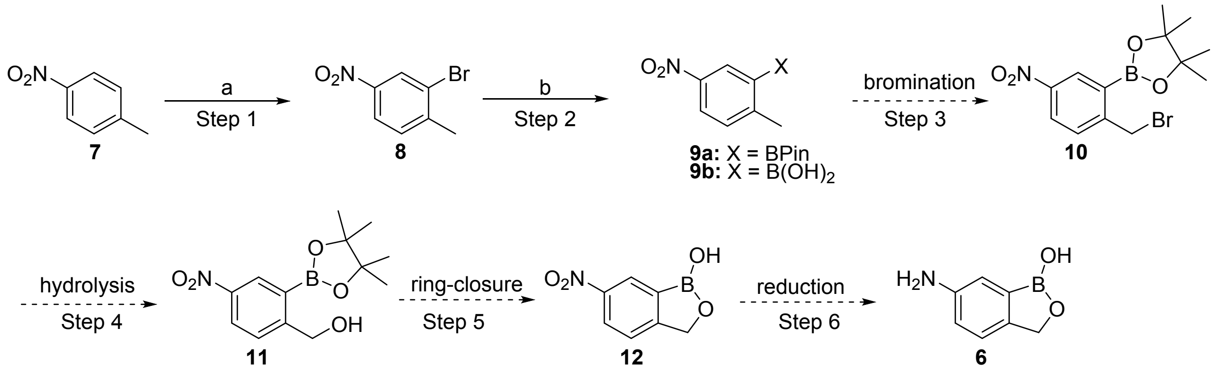

Reagents and conditions: (a) NBS, H_2SO_4, rt, 92%; (b) B_2Pin_2, 5 mol% Pd(dppf)Cl_2, KOAc, toluene, 100 °C, 90%.

Synthesis of 2-Bromo-4-nitrotoluene (8)[1]

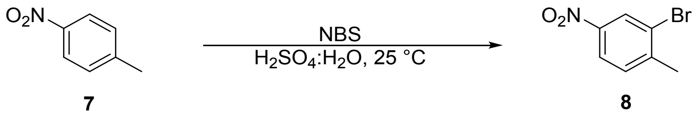

To a round bottom flask equipped with a magnetic stir bar, a mixture of p-nitrotoluene **7** (10.0 g, 1.0 eq. 72.9 mmol) and aqueous sulfuric acid (80 mL, 1:1 ratio by volume of conc. H_2SO_4 and water) were added. The flask was wrapped with aluminum foil so that darkness might prevent competitive radical reactions. The mixture was stirred for 10 minutes, then N-bromosuccinimide (15.5 g, 1.2 eq. 87.5 mmol)

was added slowly (portion wise, over 20 minutes). The mixture was stirred at room temperature for 24 h. After completion (monitored by HPLC), the product was extracted by EtOAc (50 mL x 3). The combined organic phase was washed with brine and dried over anhydrous sodium sulfate. Solvent was removed to afford 2-Bromo-4-nitrotoluene **8** (14.42 g, 92% yield) as a yellow solid.

^{1}H NMR (600 MHz, $CDCl_3$) δ/ppm: 8.37 (s, 1H), 8.05 (d, *J* = 7.8 Hz, 1H), 7.39 (d, *J* = 8.2 Hz, 1H), 2.49 (s, 3H).

^{13}C NMR (150 MHz, $CDCl_3$) δ/ppm: 146.7, 146.0, 131.2, 127.5, 125.1, 122.3, 23.4.

Melting Point 74-76 °C.

Synthesis of 4,4,5,5-tetramethyl-2-(2-methyl-5-nitrophenyl)-1,3,2-dioxaborolane (9a):

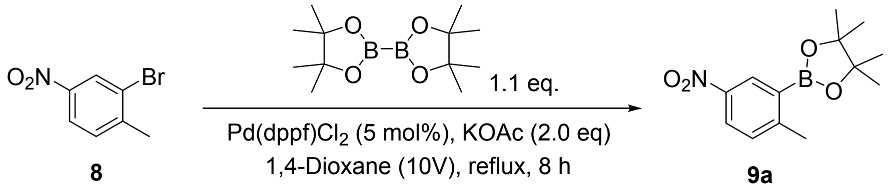

A mixture of Pd(dppf)Cl_2 (0.34 g, 5 mol%, 462.9 µmol), 2-bromo-1-methyl-4-nitrobenzene **8** (2.0 g, 1.0 eq, 9.2 mmol), bis(pinacolato)diboron (2.58 g, 1.1 eq, 10.2 mmol), and potassium acetate (1.82 g, 2.0 eq, 18.5 mmol) in 1,4-dioxane (20 mL) was degassed with N_2 at rt for 5 min, then the mixture was refluxed under nitrogen for 8 h. After completion (monitored by HPLC), the mixture was extracted with EtOAc (20 mL x 3). The organic layers were combined and washed sequentially with water and brine. The organic [1]phase was separated and dried over anhydrous Na_2SO_4, and evaporated to dryness by rotary evaporation. The residue was stirred in hexanes (20 mL) for 30 min at rt, then the solid was collected by filtration and washed with hexanes (10 mL x 3) to afford a pure compound **9a**, 2.1 g, 86% yield.

^{1}H NMR (600 MHz, $CDCl_3$) δ/ppm: 8.59 (d, *J* = 2.6 Hz, 1H), 8.12 (dd, *J* = 8.4, 2.6 Hz, 1H), 7.29 (d, *J* = 8.4 Hz, 1H), 2.62 (s, 3H), 1.35 (s, 12H).

^{13}C NMR (150 MHz, $CDCl_3$) δ/ppm: 152.9, 145.8, 130.8, 125.5, 84.4, 25.0, 22.6.

MS (m/z) (M+H): calc. for $C_{13}H_{19}BNO_4$ 264, found 264.

Melting Point 89-92 °C.

---

[1]Wagner, P. J.; Wang, L. Electronic Effects of Ring Substituents on Triplet Benzylic Biradicals. *Org. Lett.* **2006**, *8*, 645–647.

General procedure for the synthesis of diazonium salt (14) for DSC/TGA studies

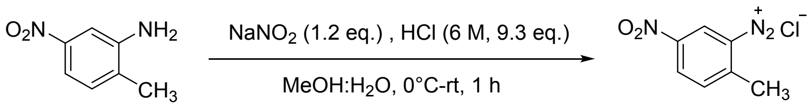

13 **14**

To an ice-cold solution of 2-methyl-5-nitroaniline **13** (1.0 g, 1.0 eq, 6.8 mmol) in methanol (20 mL) was added aq. HCl (10.2 mL, 6. 0 M, 9.3 eq, 61.1 mmol). To this mixture was added a solution of sodium nitrite (0.59 g, 1.3 eq, 8.5 mmol) in water (10 mL) dropwise using addition funnel. The resulting mixture was stirred at 0 °C for 1h (Monitored by TLC, HPLC). After completion, the solid was collected by filtration and washed with methanol to get the pure diazonium salt as a brown solid **14** (1.29g, 98%).

^{1}H NMR (600 MHz, CD_3OD) δ/ppm: 9.64 (d, J = 2.3 Hz, 1H), 8.93 (dd, J = 8.7, 2.3 Hz, 1H), 8.15 (d, J = 8.7 Hz, 1H), 2.98 (s, 3H).

^{13}C NMR (150 MHz, CD_3OD) δ/ppm: 141.9, 138.1, 126.1, 125.9, 119.4, 109.3, 9.6.

HRMS (ESI) *m/z*: $[M]^+$ Calcd for $C_7H_6N_3O_2^+$ 164.0455; Found 164.0456.

Melting Point 94-96 °C.

DSC and TGA studies of Diazonium salt 14:

Instrument: TA SDT 650

Sample holder: TA Alumina 90 uL sample cup

Scan temperature range: 30 ˚C to 250 ˚C

Scan rate: 10 ˚C/min

Purge: Nitrogen, 100 mL/min

For the DSC-TGA analysis, samples were analyzed as received in alumina 90 μL sample cups. Approximately 5 mg of sample was added to the sample cup after tearing. The thermogram ran from 30-250 ˚C at a ramp of 10 ˚C/min.

DSC **TGA**

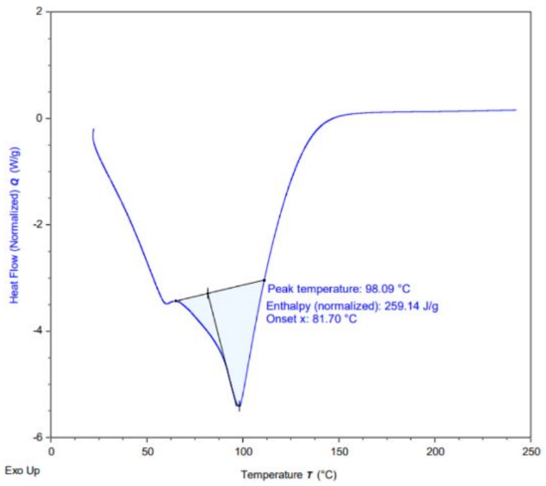

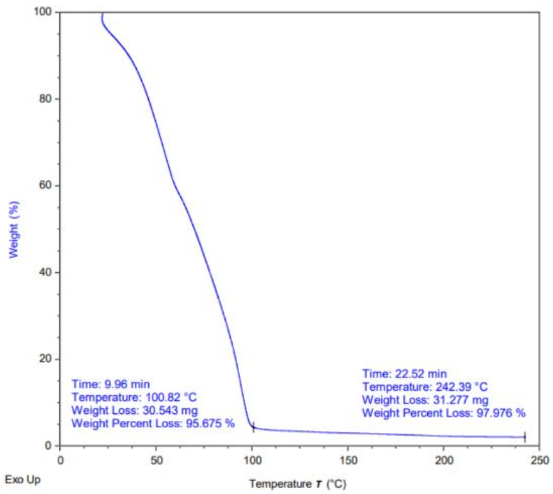

Figure S1. DSC (left) and TGA (right) results of the diazonium salt collected directly from a reaction mixture.

DSC **TGA**

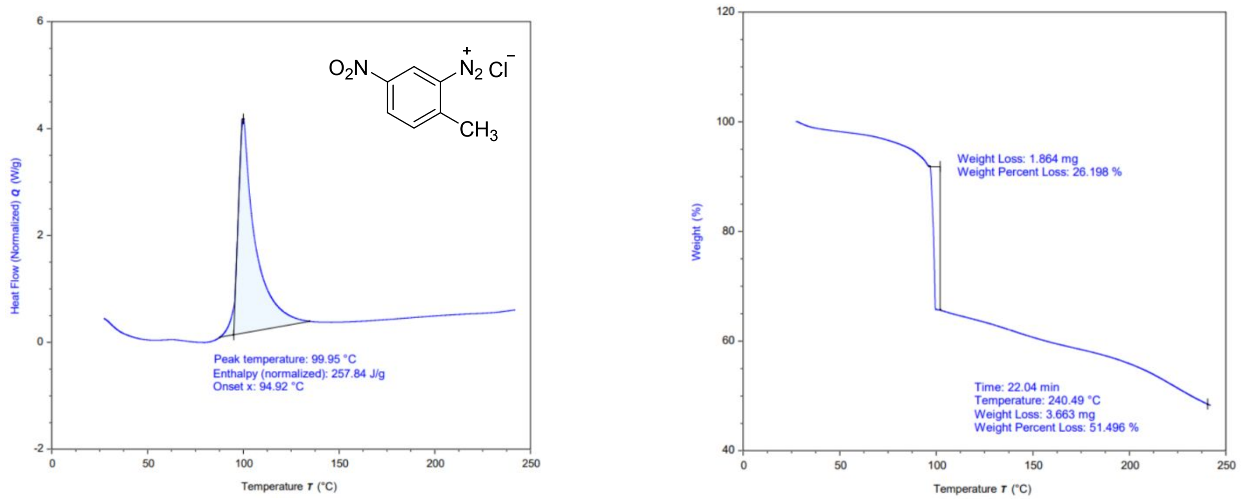

Figure S2. DSC (left) and TGA (right) results of the purified diazonium salt **14**.

^{1}H and ^{13}C NMR Spectra of all compounds

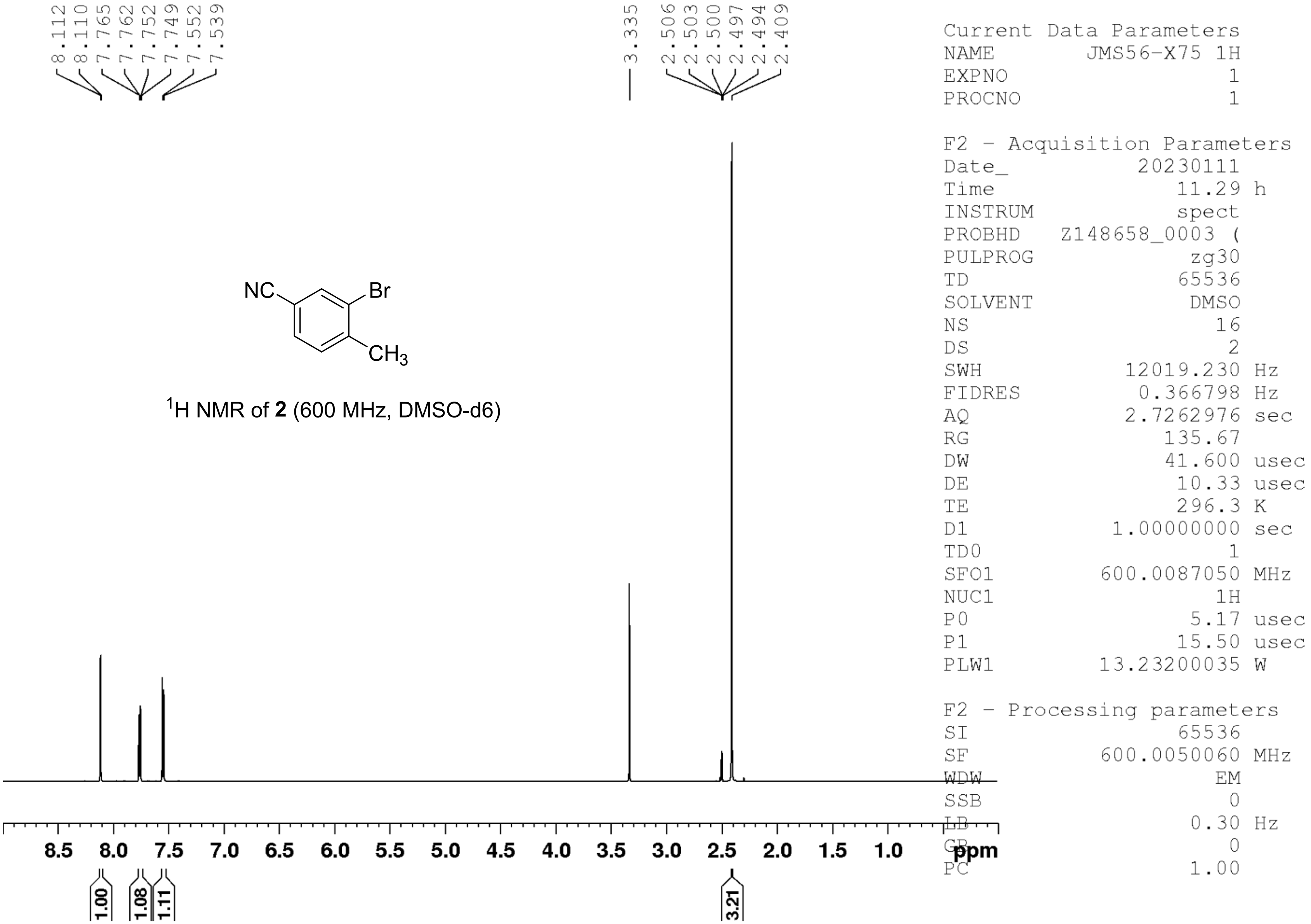

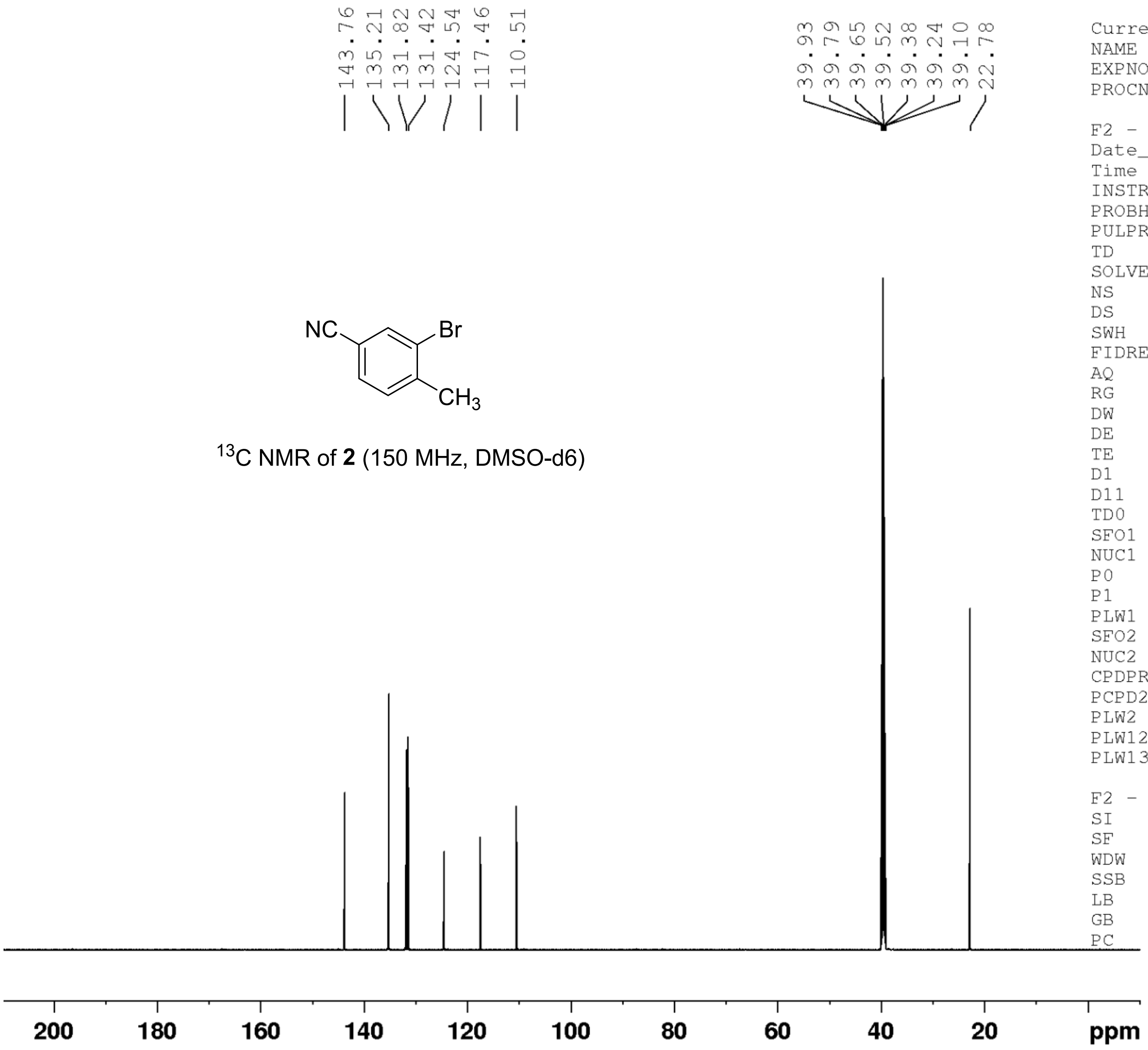

^{13}C NMR of **2** (150 MHz, DMSO-d6)

```
Current Data Parameters
NAME       JMS56-X75 13C
EXPNO                  1
PROCNO                 1

F2 - Acquisition Parameters
Date_           20230112
Time                5.29 h
INSTRUM             spect
PROBHD    Z148658_0003 (
PULPROG           zgpg30
TD                 65536
SOLVENT             DMSO
NS                  5000
DS                     4
SWH            36231.883 Hz
FIDRES          1.105709 Hz
AQ             0.9043968 sec
RG                199.73
DW                13.800 usec
DE                  6.50 usec
TE                 298.1 K
D1            2.00000000 sec
D11           0.03000000 sec
TD0                    1
SFO1         150.8864644 MHz
NUC1                 13C
P0                  4.00 usec
P1                 12.00 usec
PLW1         77.65699768 W
SFO2         600.0074000 MHz
NUC2                  1H
CPDPRG[2         waltz65
PCPD2              70.00 usec
PLW2         13.23200035 W
PLW12         0.64876997 W
PLW13         0.32633001 W

F2 - Processing parameters
SI                 32768
SF           150.8714518 MHz
WDW                   EM
SSB                    0
LB                  1.00 Hz
GB                     0
PC                  1.40
```

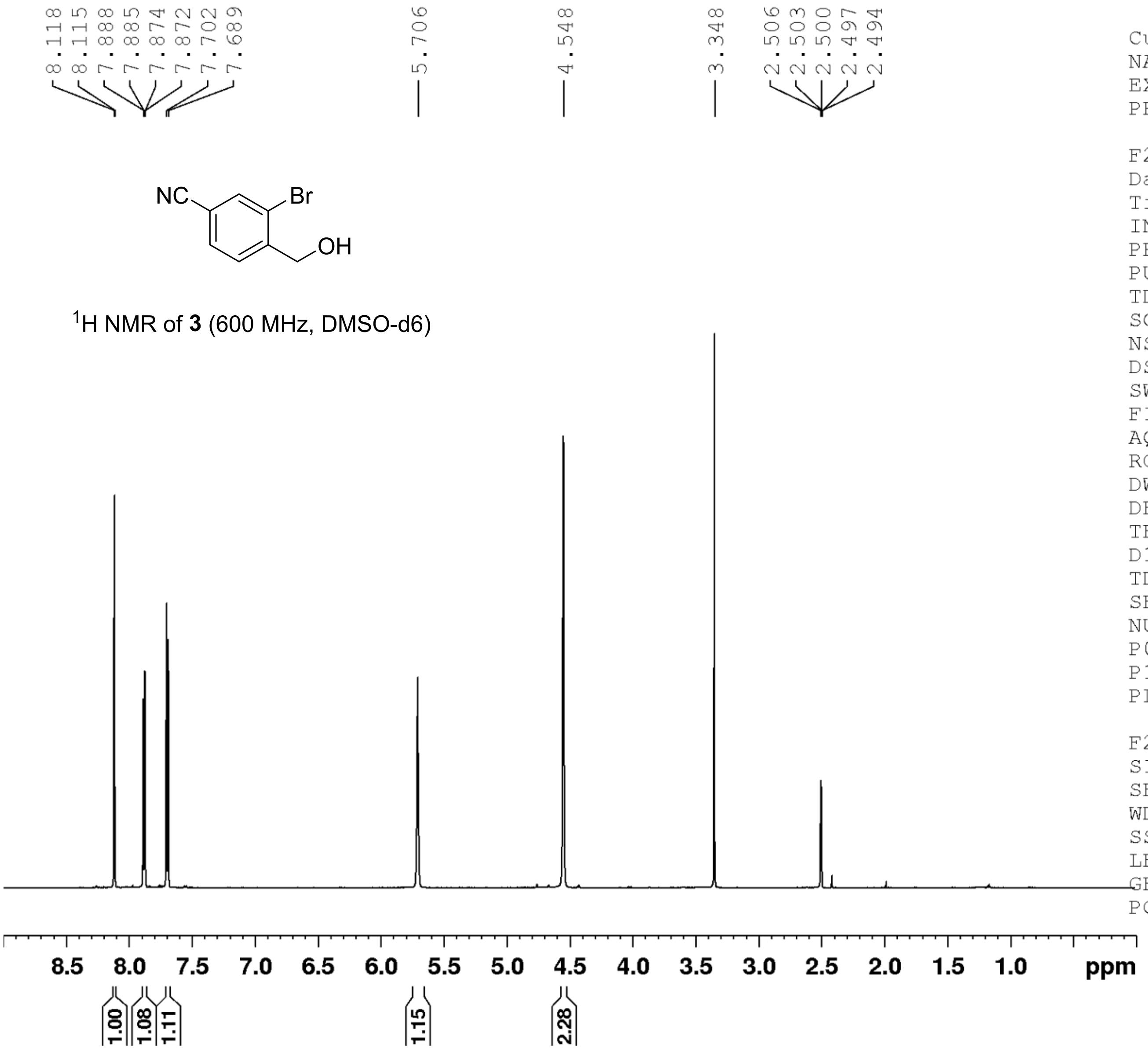

^{1}H NMR of **3** (600 MHz, DMSO-d6)

```
Current Data Parameters
NAME        JMS56-X77 1H
EXPNO                  1
PROCNO                 1

F2 - Acquisition Parameters
Date_           20230125
Time               11.22 h
INSTRUM            spect
PROBHD    Z148658_0003 (
PULPROG             zg30
TD                 65536
SOLVENT             DMSO
NS                    16
DS                     2
SWH            12019.230 Hz
FIDRES          0.366798 Hz
AQ             2.7262976 sec
RG                135.67
DW                41.600 usec
DE                 10.33 usec
TE                 296.4 K
D1            1.00000000 sec
TD0                    1
SFO1         600.0087050 MHz
NUC1                  1H
P0                  5.17 usec
P1                 15.50 usec
PLW1         13.23200035 W

F2 - Processing parameters
SI                 65536
SF           600.0050060 MHz
WDW                   EM
SSB                    0
LB                  0.30 Hz
GB                     0
PC                  1.00
```

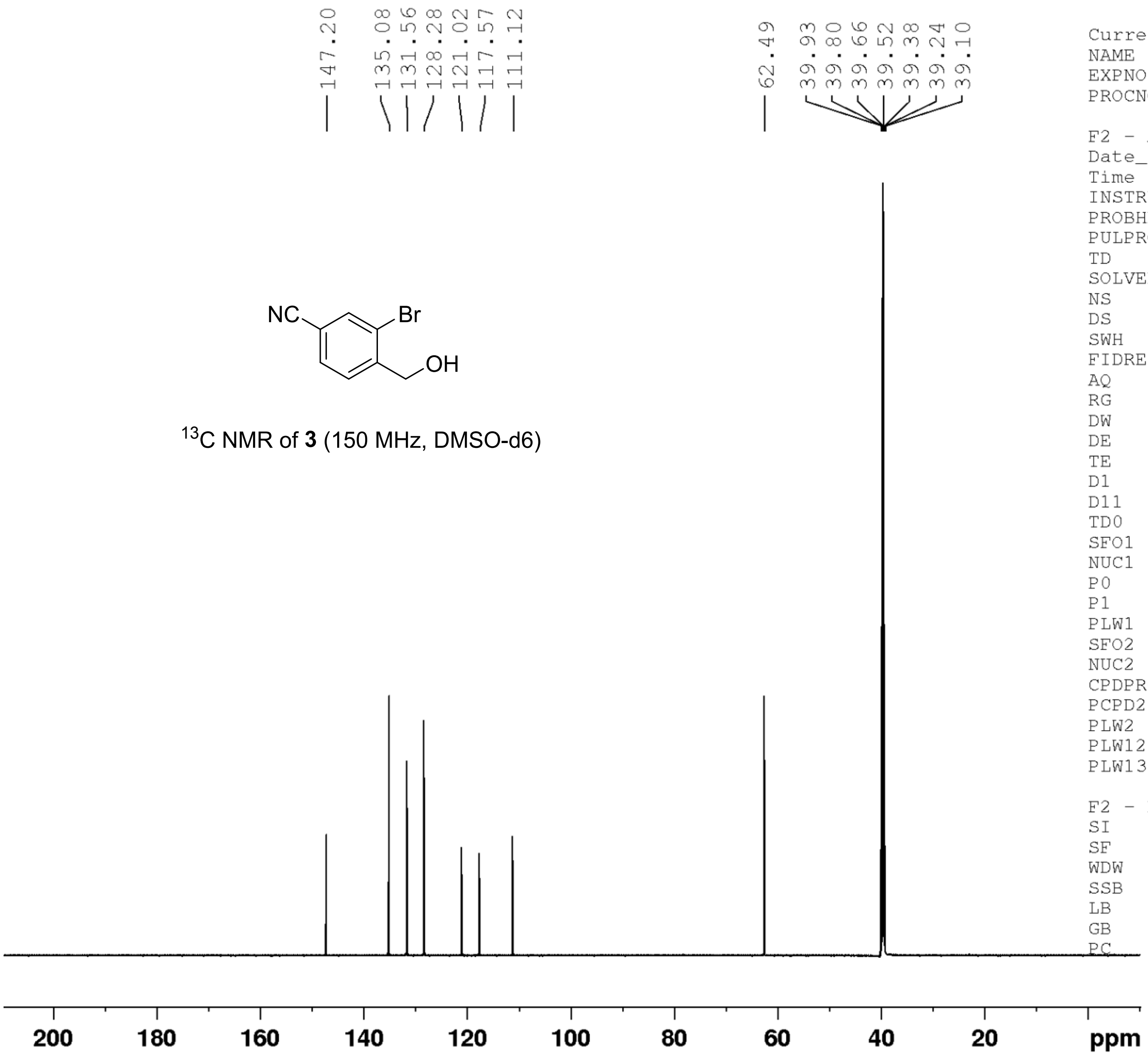

^{13}C NMR of **3** (150 MHz, DMSO-d6)

Current Data Parameters
NAME JMS56-X77 13C
EXPNO 1
PROCNO 1

F2 - Acquisition Parameters
Date_ 20230126
Time 2.55 h
INSTRUM spect
PROBHD Z148658_0003 (
PULPROG zgpg30
TD 65536
SOLVENT DMSO
NS 5000
DS 4
SWH 36231.883 Hz
FIDRES 1.105709 Hz
AQ 0.9043968 sec
RG 199.73
DW 13.800 usec
DE 6.50 usec
TE 297.6 K
D1 2.00000000 sec
D11 0.03000000 sec
TD0 1
SFO1 150.8864644 MHz
NUC1 13C
P0 4.00 usec
P1 12.00 usec
PLW1 77.65699768 W
SFO2 600.0074000 MHz
NUC2 1H
CPDPRG[2 waltz65
PCPD2 70.00 usec
PLW2 13.23200035 W
PLW12 0.64876997 W
PLW13 0.32633001 W

F2 - Processing parameters
SI 32768
SF 150.8714484 MHz
WDW EM
SSB 0
LB 1.00 Hz
GB 0
PC 1.40

^{1}H NMR of **4** (600 MHz, DMSO-d6)

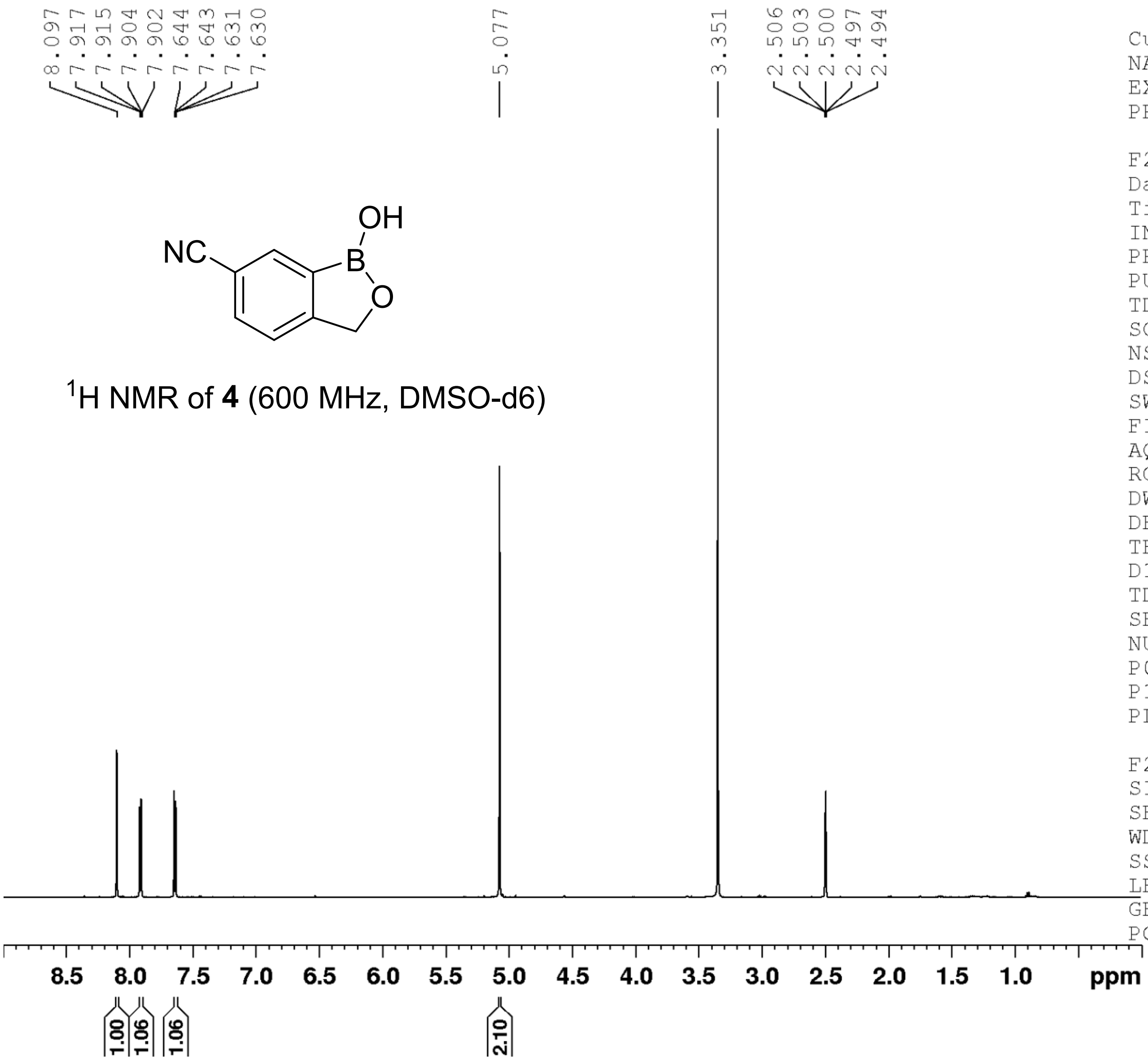

Current Data Parameters

NAME	JMS56-X80 1H
EXPNO	1
PROCNO	1

F2 - Acquisition Parameters

Date_	20230206
Time	10.16 h
INSTRUM	spect
PROBHD	Z148658_0003 (
PULPROG	zg30
TD	65536
SOLVENT	DMSO
NS	16
DS	2
SWH	12019.230 Hz
FIDRES	0.366798 Hz
AQ	2.7262976 sec
RG	199.73
DW	41.600 usec
DE	10.33 usec
TE	296.4 K
D1	1.00000000 sec
TD0	1
SFO1	600.0087050 MHz
NUC1	1H
P0	5.17 usec
P1	15.50 usec
PLW1	13.23200035 W

F2 - Processing parameters

SI	65536
SF	600.0050061 MHz
WDW	EM
SSB	0
LB	0.30 Hz
GB	0
PC	1.00

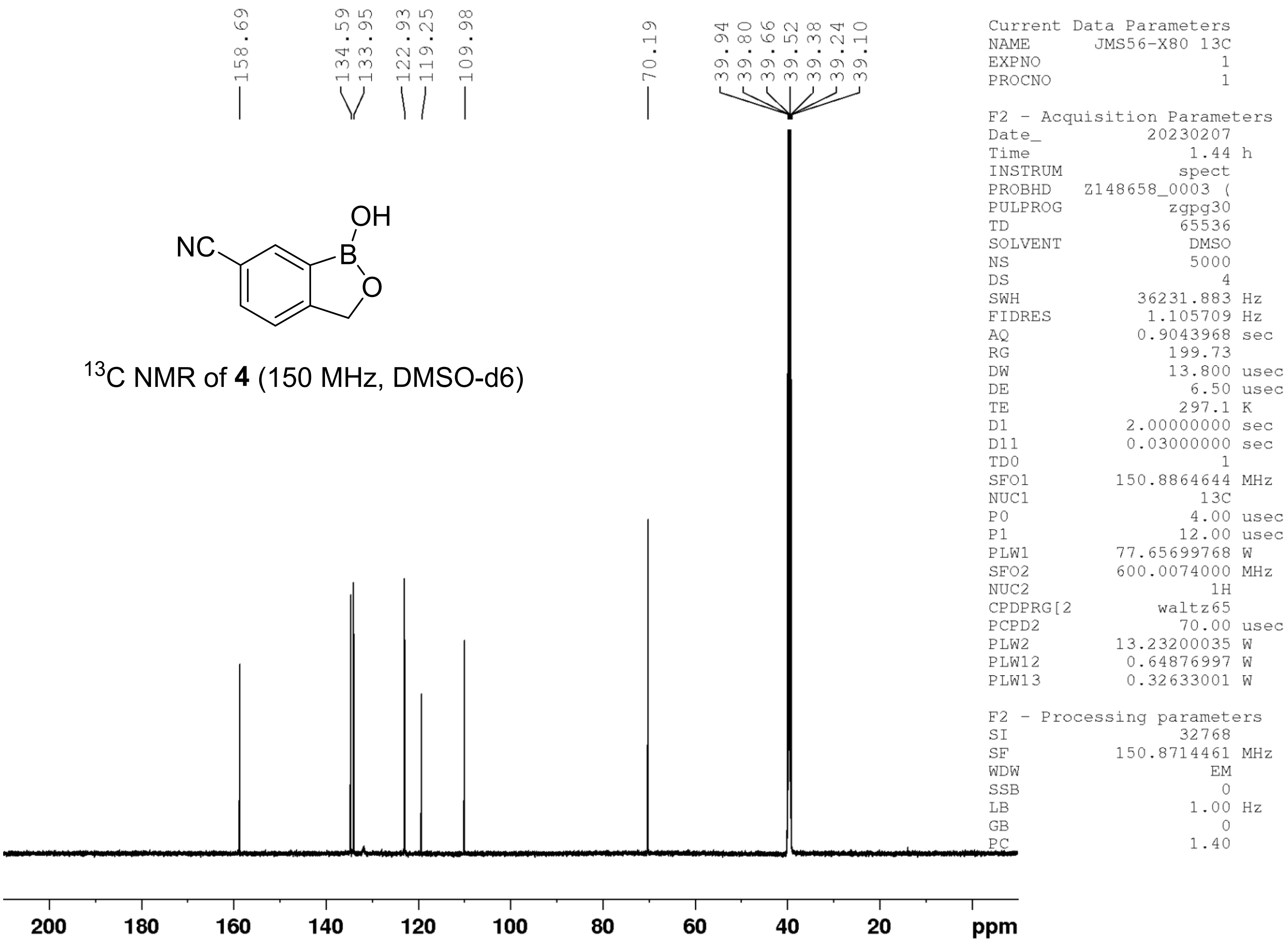

^{13}C NMR of **4** (150 MHz, DMSO-d6)

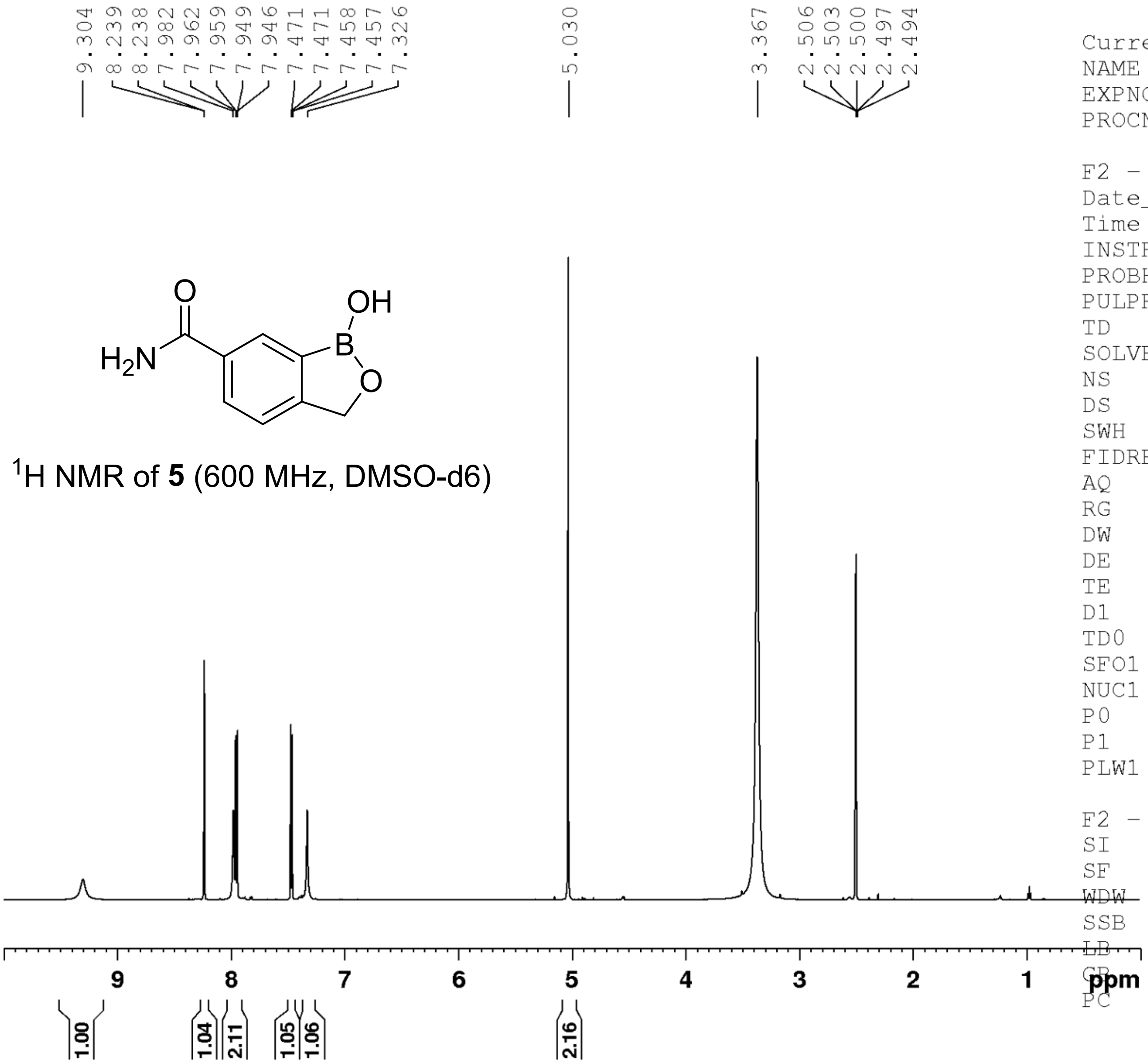

^{1}H NMR of **5** (600 MHz, DMSO-d6)

Current Data Parameters
NAME JMS56-X81 1H
EXPNO 3
PROCNO 1

F2 - Acquisition Parameters
Date_ 20230228
Time 12.31 h
INSTRUM spect
PROBHD Z855801_0105 (
PULPROG zg30
TD 65536
SOLVENT DMSO
NS 16
DS 2
SWH 12019.230 Hz
FIDRES 0.366798 Hz
AQ 2.7262976 sec
RG 110.56
DW 41.600 usec
DE 10.93 usec
TE 295.1 K
D1 1.00000000 sec
TD0 1
SFO1 599.9587047 MHz
NUC1 1H
P0 4.00 usec
P1 12.00 usec
PLW1 15.77700043 W

F2 - Processing parameters
SI 65536
SF 599.9550063 MHz
WDW EM
SSB 0
LB 0.30 Hz
GB 0
PC 1.00

^{13}C NMR of **5** (150 MHz, DMSO-d6)

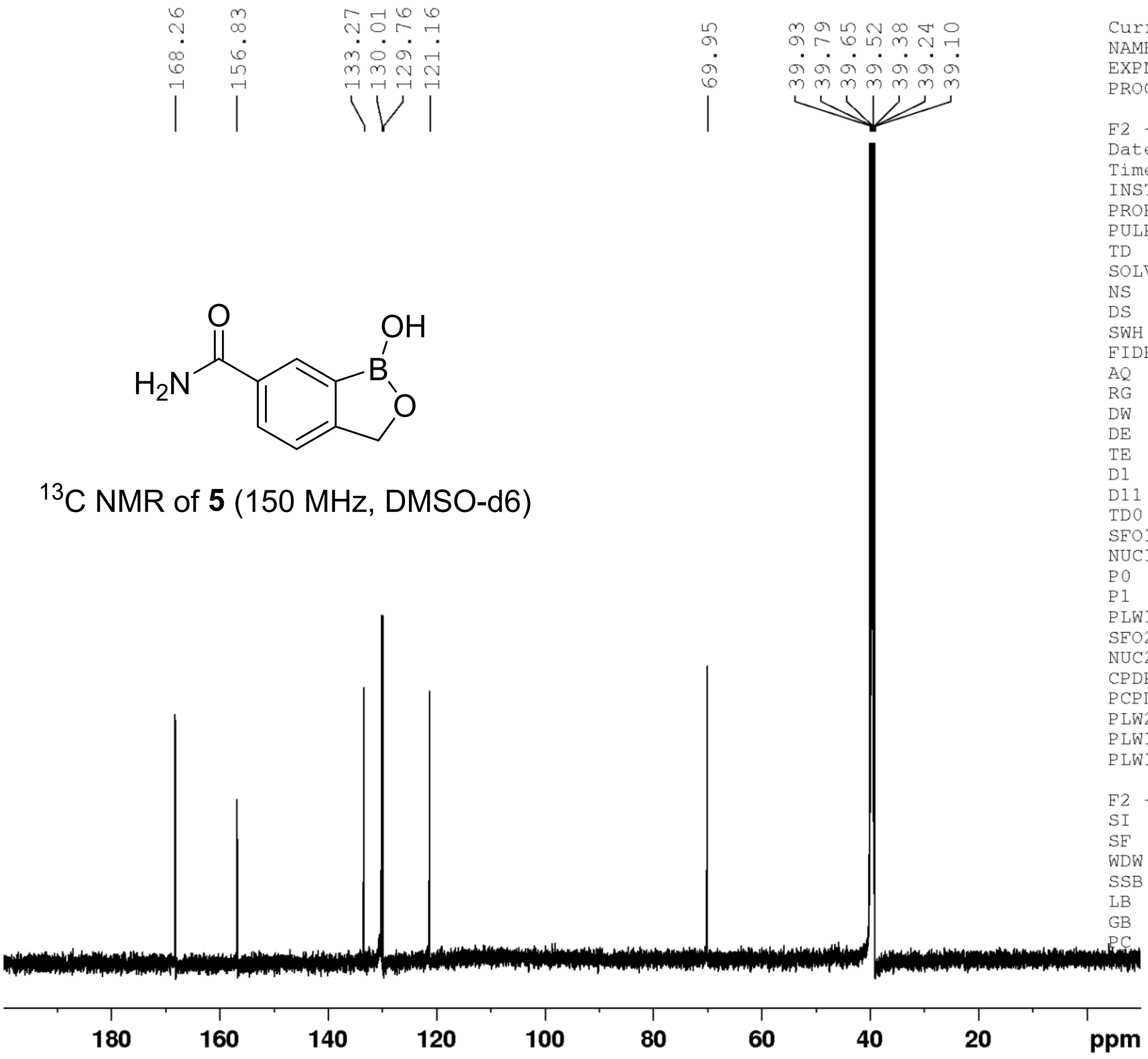


```
Current Data Parameters
NAME      JMS56-X81 13C
EXPNO                 2
PROCNO                1

F2 - Acquisition Parameters
Date_          20230302
Time               2.52 h
INSTRUM           spect
PROBHD   Z855801_0105 (
PULPROG         zgpg30
TD                65536
SOLVENT            DMSO
NS                 5000
DS                    4
SWH           36231.883 Hz
FIDRES         1.105709 Hz
AQ            0.9043968 sec
RG               194.75
DW               13.800 usec
DE                 6.50 usec
TE                296.6 K
D1           2.00000000 sec
D11          0.03000000 sec
TD0                   1
SFO1        150.8738906 MHz
NUC1                13C
P0                 3.67 usec
P1                11.00 usec
PLW1       178.30999756 W
SFO2        599.9573998 MHz
NUC2                 1H
CPDPRG[2        waltz65
PCPD2             70.00 usec
PLW2        15.77700043 W
PLW12        0.46366000 W
PLW13        0.23322000 W

F2 - Processing parameters
SI                32768
SF          150.8588751 MHz
WDW                  EM
SSB                   0
LB                 1.00 Hz
GB                    0
PC                 1.40
```

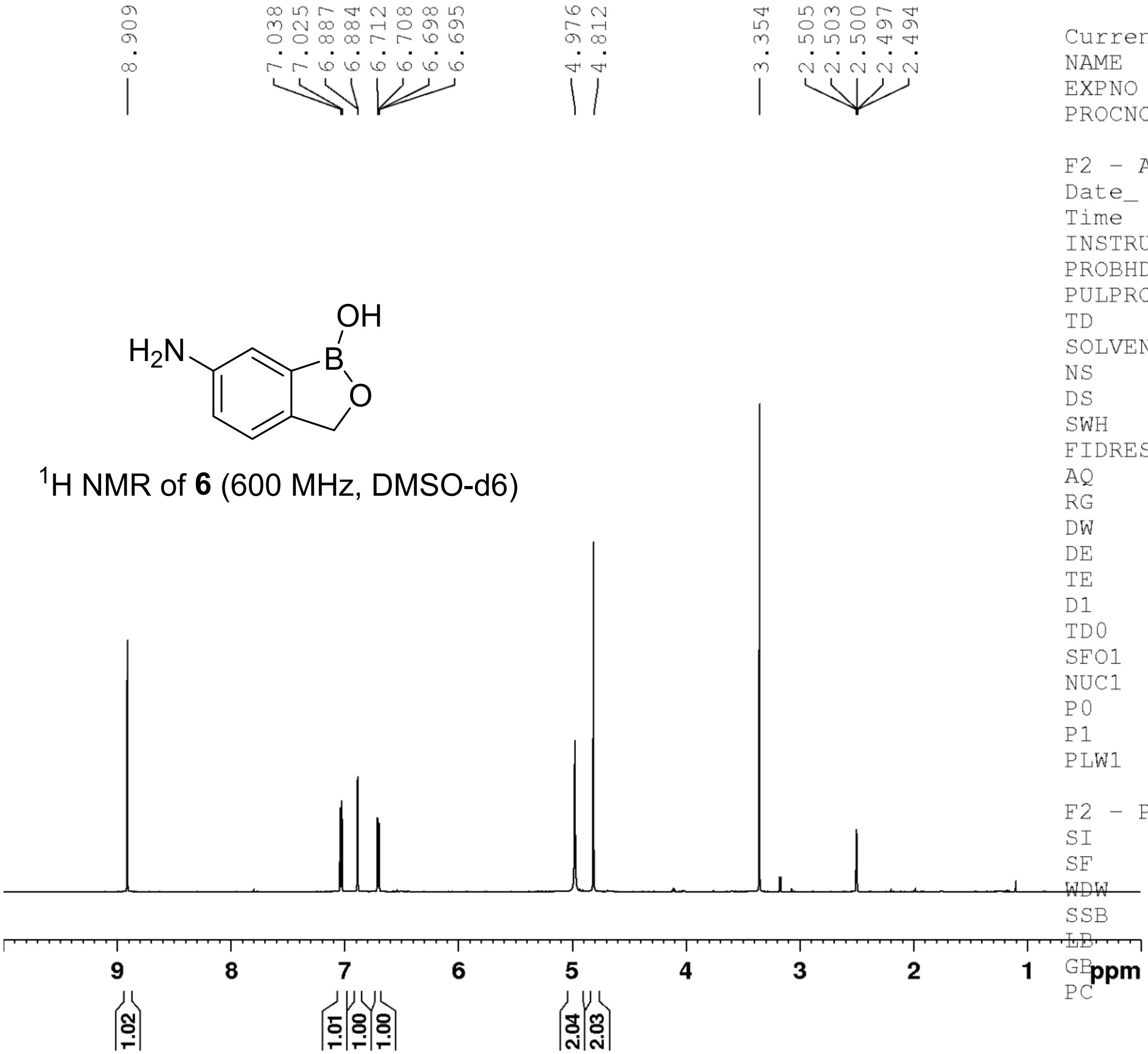

^{1}H NMR of **6** (600 MHz, DMSO-d6)

Current Data Parameters
NAME JMS56-X74 1H
EXPNO 1
PROCNO 1

F2 - Acquisition Parameters
Date_ 20230221
Time 13.18 h
INSTRUM spect
PROBHD Z148658_0003 (
PULPROG zg30
TD 65536
SOLVENT DMSO
NS 16
DS 2
SWH 12019.230 Hz
FIDRES 0.366798 Hz
AQ 2.7262976 sec
RG 199.73
DW 41.600 usec
DE 10.33 usec
TE 296.2 K
D1 1.00000000 sec
TD0 1
SFO1 600.0087050 MHz
NUC1 1H
P0 5.17 usec
P1 15.50 usec
PLW1 13.23200035 W

F2 - Processing parameters
SI 65536
SF 600.0050061 MHz
WDW EM
SSB 0
LB 0.30 Hz
GB 0
PC 1.00

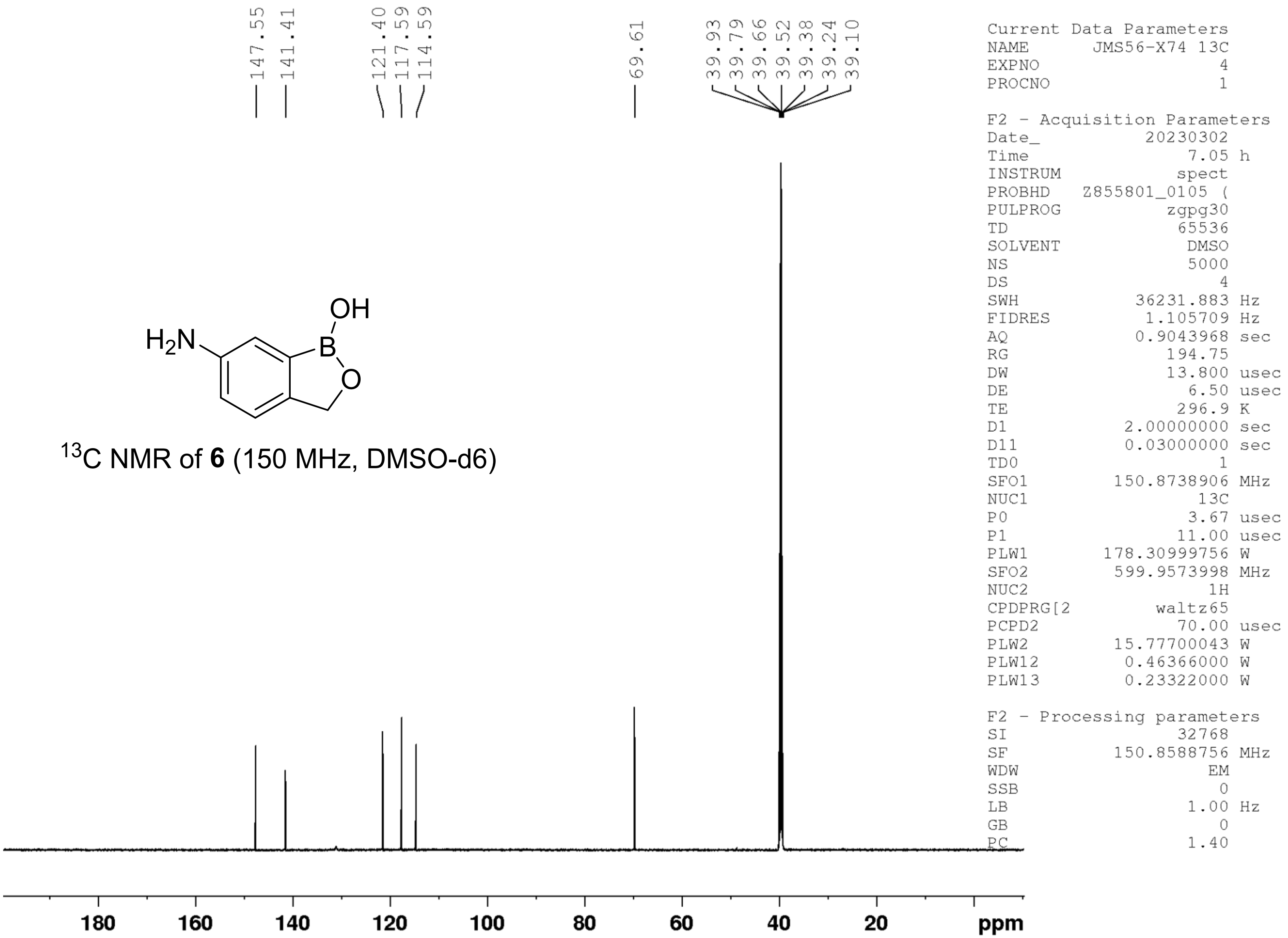

^{13}C NMR of **6** (150 MHz, DMSO-d6)

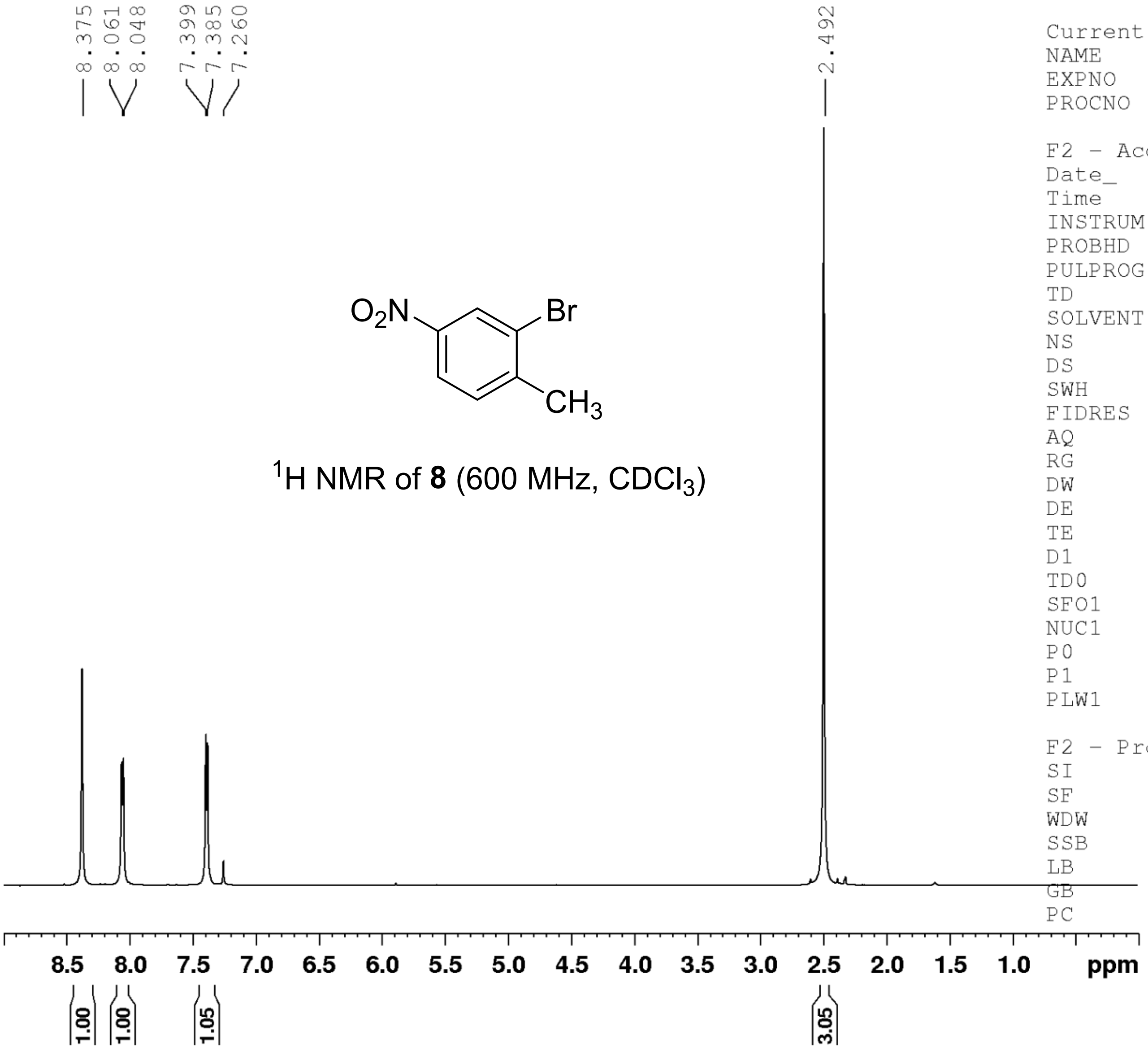

^{1}H NMR of **8** (600 MHz, $CDCl_3$)

```
Current Data Parameters
NAME      PVK49-X8 Product Pure 2nd
EXPNO                     1
PROCNO                    1

F2 - Acquisition Parameters
Date_           20230106
Time               17.35 h
INSTRUM            spect
PROBHD   Z148658_0003 (
PULPROG             zg30
TD                 65536
SOLVENT            CDCl3
NS                    16
DS                     2
SWH           12019.230 Hz
FIDRES         0.366798 Hz
AQ            2.7262976 sec
RG                 89.69
DW               41.600 usec
DE                10.33 usec
TE                296.8 K
D1           1.00000000 sec
TD0                    1
SFO1        600.0087050 MHz
NUC1                  1H
P0                  5.17 usec
P1                 15.50 usec
PLW1        13.23200035 W

F2 - Processing parameters
SI                 65536
SF          600.0050160 MHz
WDW                   EM
SSB                    0
LB                  0.30 Hz
GB                     0
PC                  1.00
```

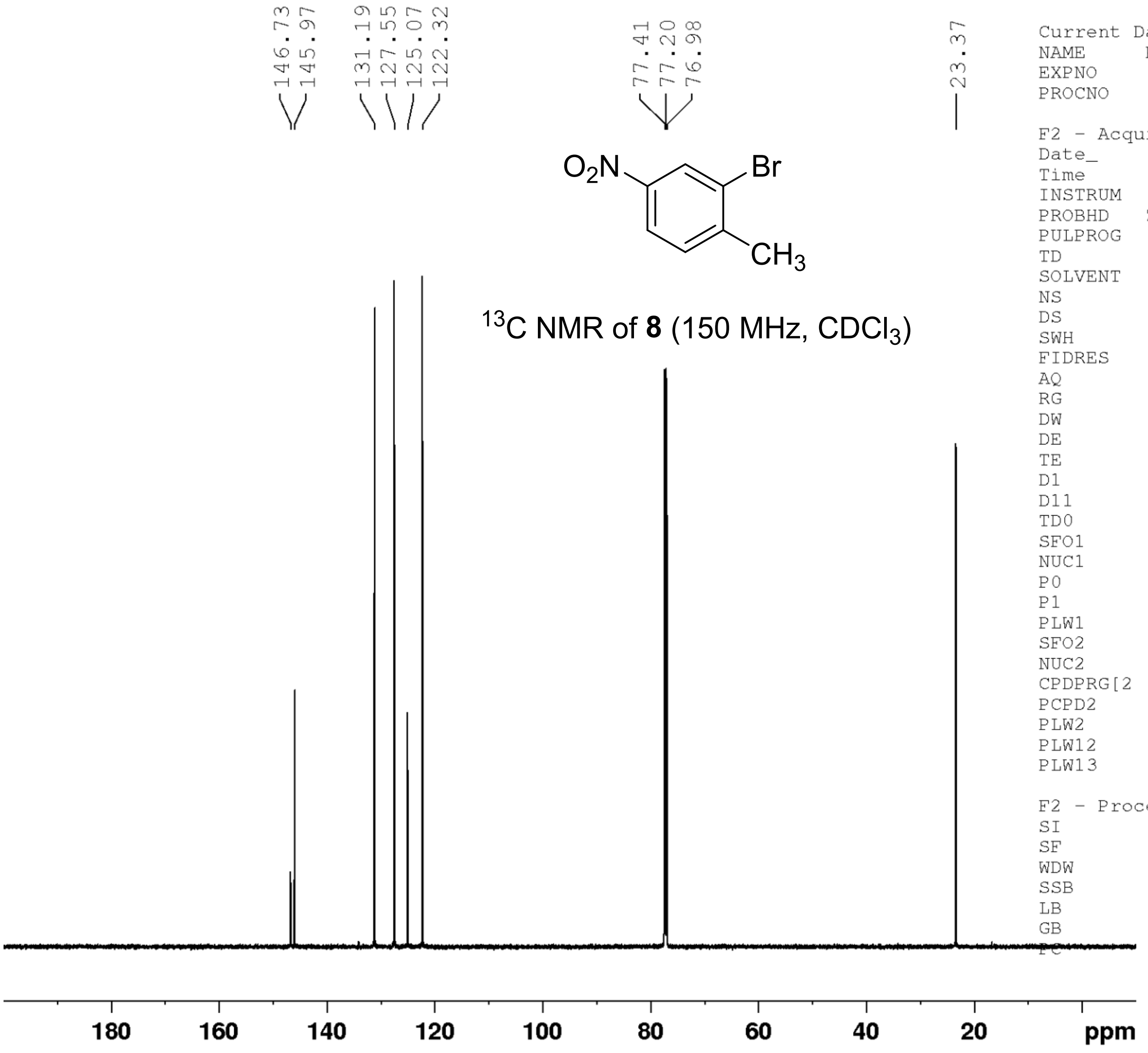

^{13}C NMR of **8** (150 MHz, $CDCl_3$)

```
Current Data Parameters
NAME     PVK49-X8 Product Pure 2nd
EXPNO                    2
PROCNO                   1

F2 - Acquisition Parameters
Date_             20230106
Time                 18.27 h
INSTRUM              spect
PROBHD   Z148658_0003 (
PULPROG            zgpg30
TD                   65536
SOLVENT              CDCl3
NS                    1024
DS                       4
SWH             36231.883 Hz
FIDRES           1.105709 Hz
AQ              0.9043968 sec
RG                  199.73
DW                  13.800 usec
DE                    6.50 usec
TE                   298.2 K
D1             2.00000000 sec
D11            0.03000000 sec
TD0                      1
SFO1          150.8864644 MHz
NUC1                   13C
P0                    4.00 usec
P1                   12.00 usec
PLW1          77.65699768 W
SFO2          600.0074000 MHz
NUC2                    1H
CPDPRG[2           waltz65
PCPD2                70.00 usec
PLW2          13.23200035 W
PLW12          0.64876997 W
PLW13          0.32633001 W

F2 - Processing parameters
SI                   32768
SF            150.8713585 MHz
WDW                     EM
SSB                      0
LB                    1.00 Hz
GB                       0
PC                    1.40
```

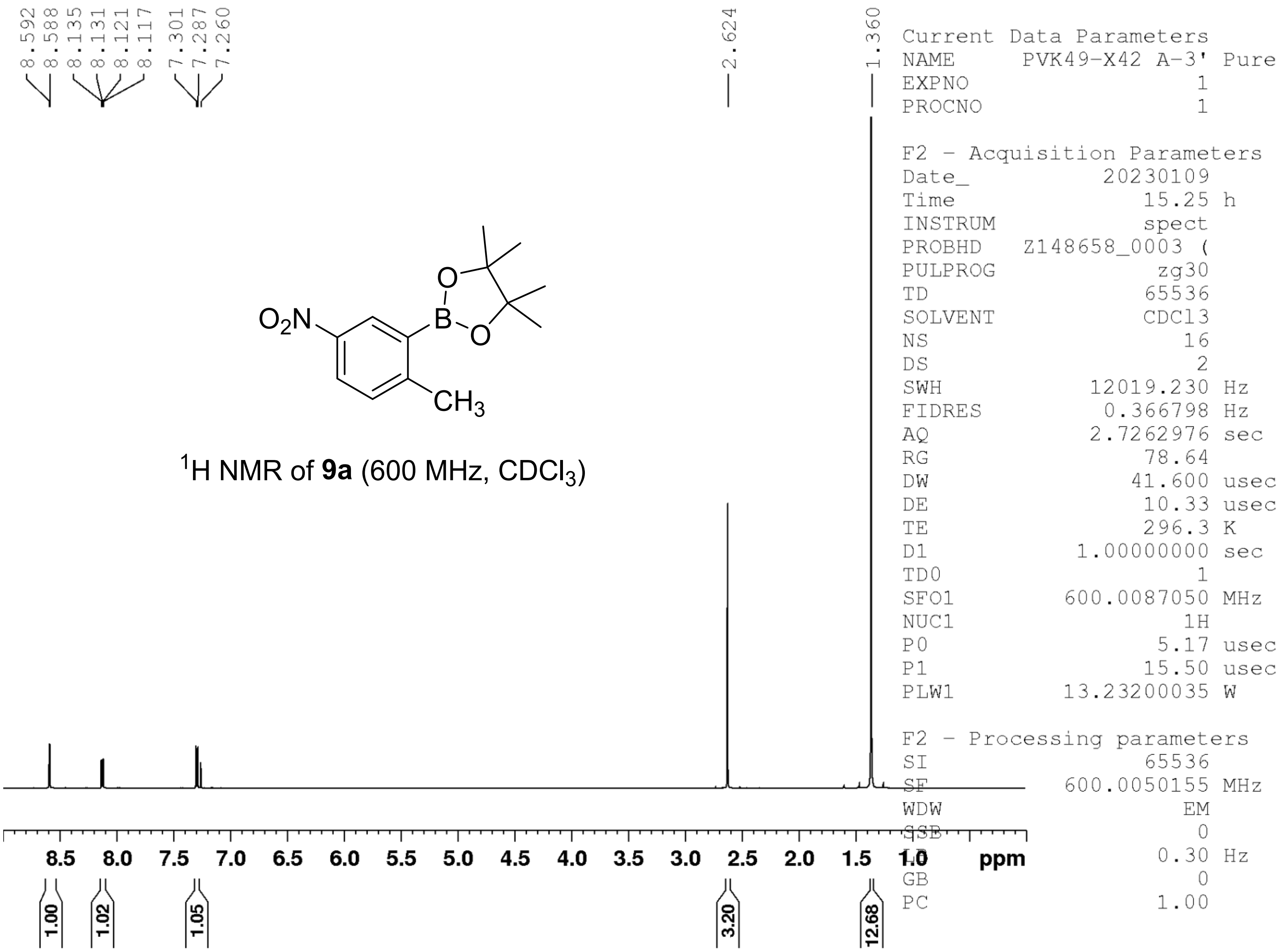

[1] H NMR of **9a** (600 MHz, $CDCl_3$)

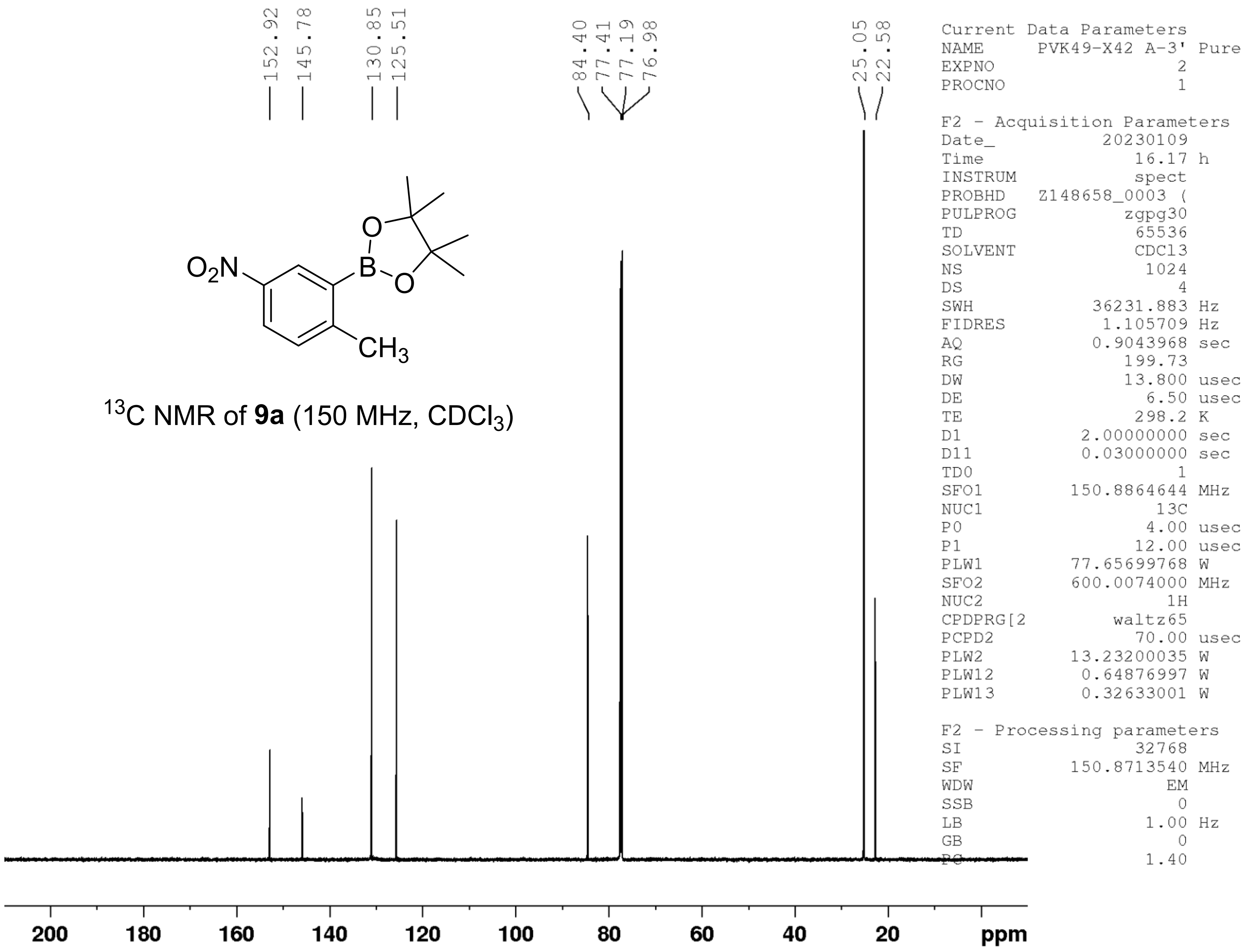

^{13}C NMR of **9a** (150 MHz, $CDCl_3$)

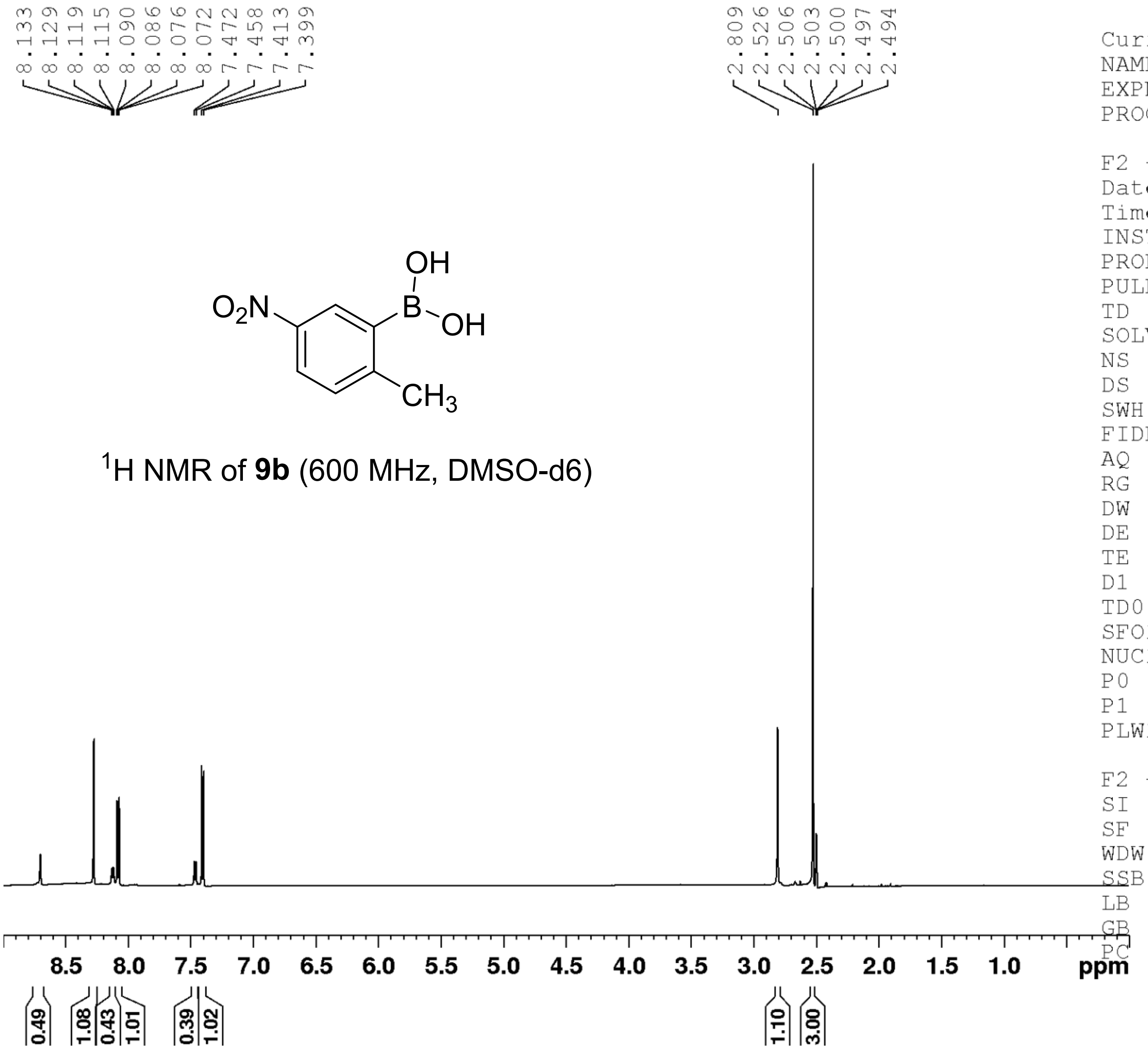

^{1}H NMR of **9b** (600 MHz, DMSO-d6)

Current Data Parameters
NAME PVK49-X65 A3 Pure
EXPNO 1
PROCNO 1

F2 - Acquisition Parameters
Date_ 20230306
Time 13.18 h
INSTRUM spect
PROBHD Z855801_0105 (
PULPROG zg30
TD 65536
SOLVENT DMSO
NS 16
DS 2
SWH 12019.230 Hz
FIDRES 0.366798 Hz
AQ 2.7262976 sec
RG 63.39
DW 41.600 usec
DE 10.93 usec
TE 295.0 K
D1 1.00000000 sec
TD0 1
SFO1 599.9587047 MHz
NUC1 1H
P0 4.00 usec
P1 12.00 usec
PLW1 15.77700043 W

F2 - Processing parameters
SI 65536
SF 599.9550061 MHz
WDW EM
SSB 0
LB 0.30 Hz
GB 0
PC 1.00

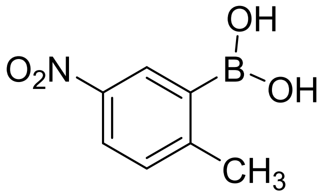

^{1}H NMR of **9b** (600 MHz, DMSO-d6)

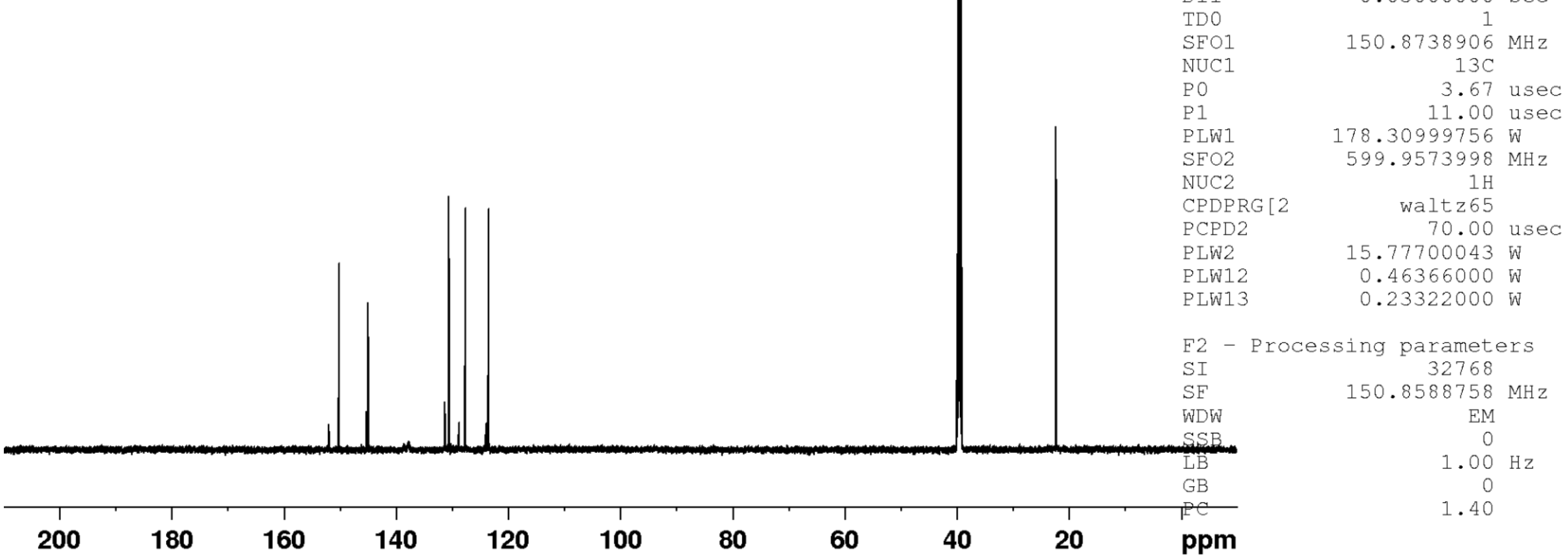

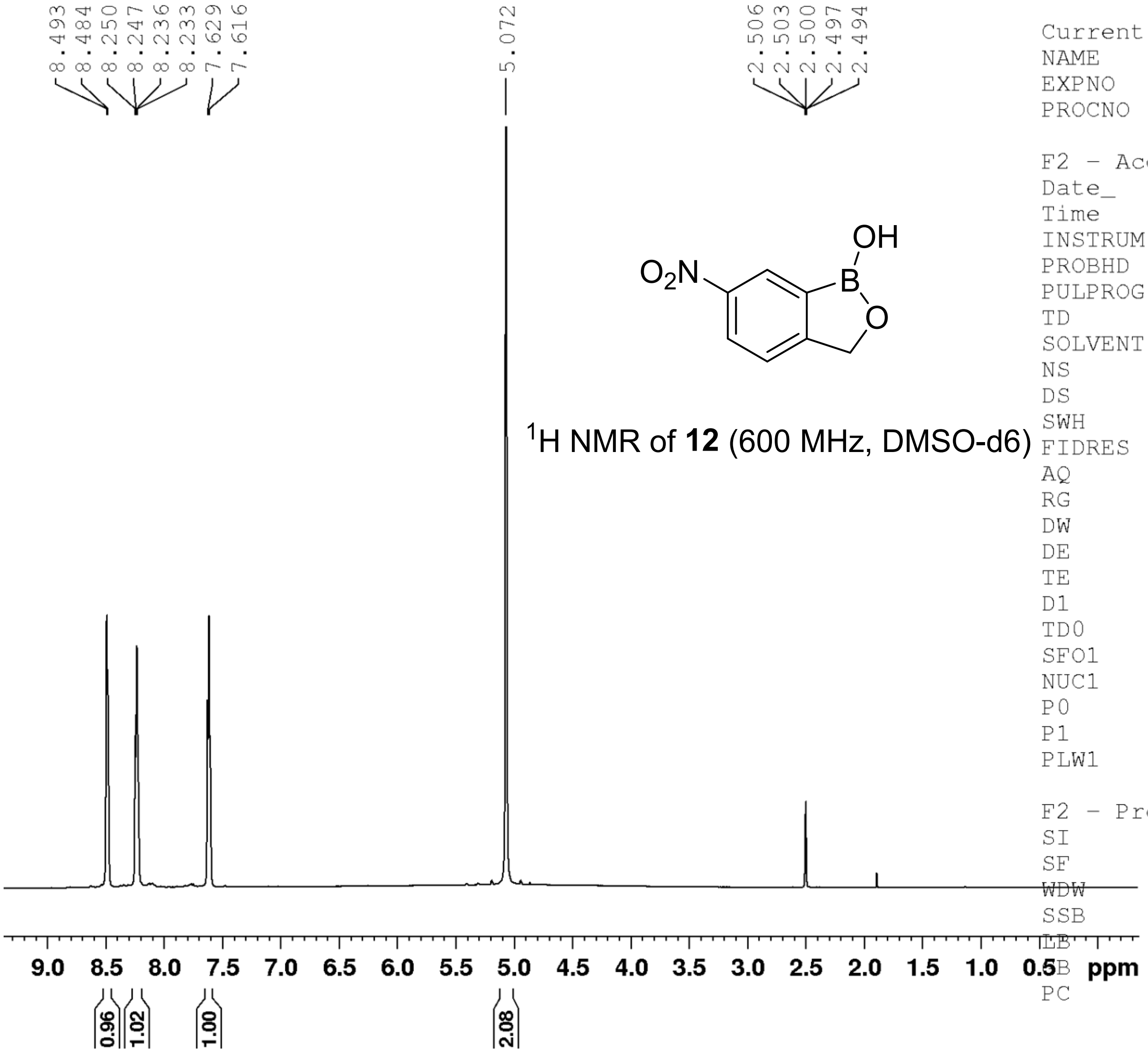

^{1}H NMR of **12** (600 MHz, DMSO-d6)

```
Current Data Parameters
NAME      PVK49-X68 Pure A-4
EXPNO                      1
PROCNO                     1

F2 - Acquisition Parameters
Date_             20230110
Time                 15.19 h
INSTRUM              spect
PROBHD    Z148658_0003 (
PULPROG               zg30
TD                   65536
SOLVENT               DMSO
NS                      16
DS                       2
SWH              12019.230 Hz
FIDRES            0.366798 Hz
AQ               2.7262976 sec
RG                      61
DW                  41.600 usec
DE                   10.33 usec
TE                   296.2 K
D1              1.00000000 sec
TD0                      1
SFO1         600.0087050 MHz
NUC1                    1H
P0                    5.17 usec
P1                   15.50 usec
PLW1          13.23200035 W

F2 - Processing parameters
SI                   65536
SF           600.0050064 MHz
WDW                     EM
SSB                      0
LB                    0.30 Hz
GB                       0
PC                    1.00
```

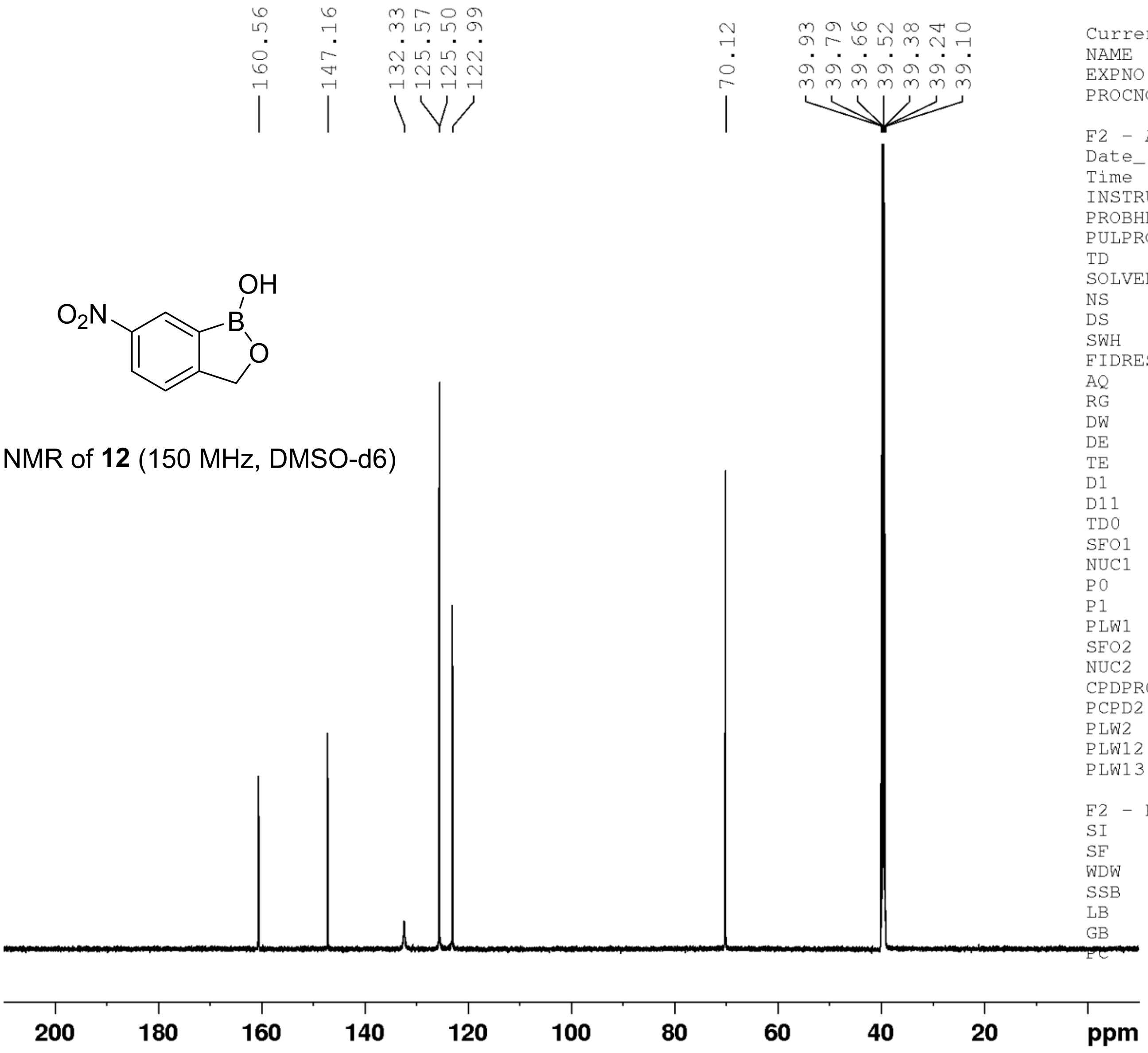

^{13}C NMR of **12** (150 MHz, DMSO-d6)

```
Current Data Parameters
NAME      PVK49-X68 Pure A-4
EXPNO                      2
PROCNO                     1

F2 - Acquisition Parameters
Date_             20230110
Time                 16.11 h
INSTRUM              spect
PROBHD    Z148658_0003 (
PULPROG            zgpg30
TD                  65536
SOLVENT              DMSO
NS                   1024
DS                      4
SWH             36231.883 Hz
FIDRES           1.105709 Hz
AQ              0.9043968 sec
RG                 199.73
DW                 13.800 usec
DE                   6.50 usec
TE                  298.1 K
D1             2.00000000 sec
D11            0.03000000 sec
TD0                     1
SFO1          150.8864644 MHz
NUC1                  13C
P0                   4.00 usec
P1                  12.00 usec
PLW1          77.65699768 W
SFO2          600.0074000 MHz
NUC2                   1H
CPDPRG[2          waltz65
PCPD2               70.00 usec
PLW2          13.23200035 W
PLW12          0.64876997 W
PLW13          0.32633001 W

F2 - Processing parameters
SI                  32768
SF            150.8714319 MHz
WDW                    EM
SSB                     0
LB                   1.00 Hz
GB                      0
PC                   1.40
```

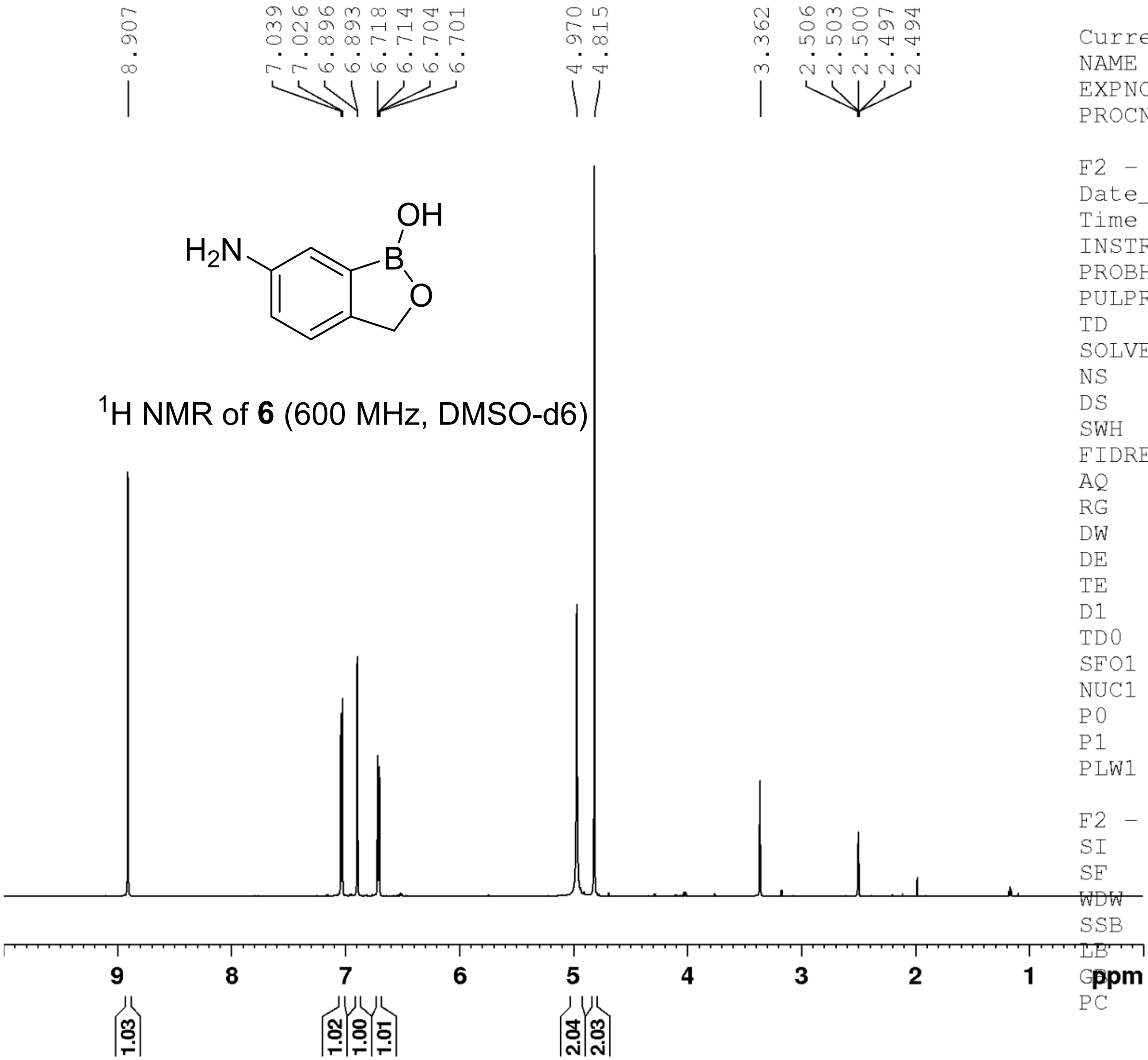

^{1}H NMR of **6** (600 MHz, DMSO-d6)

Current Data Parameters
NAME PVK49-X78 A-5 Pure_2
EXPNO 1
PROCNO 1

F2 - Acquisition Parameters
Date_ 20230111
Time 16.33 h
INSTRUM spect
PROBHD Z148658_0003 (
PULPROG zg30
TD 65536
SOLVENT DMSO
NS 16
DS 2
SWH 12019.230 Hz
FIDRES 0.366798 Hz
AQ 2.7262976 sec
RG 106.9
DW 41.600 usec
DE 10.33 usec
TE 298.0 K
D1 1.00000000 sec
TD0 1
SFO1 600.0087050 MHz
NUC1 1H
P0 5.17 usec
P1 15.50 usec
PLW1 13.23200035 W

F2 - Processing parameters
SI 65536
SF 600.0050063 MHz
WDW EM
SSB 0
LB 0.30 Hz
GB 0
PC 1.00

^{13}C NMR of **6** (150 MHz, DMSO-d6)

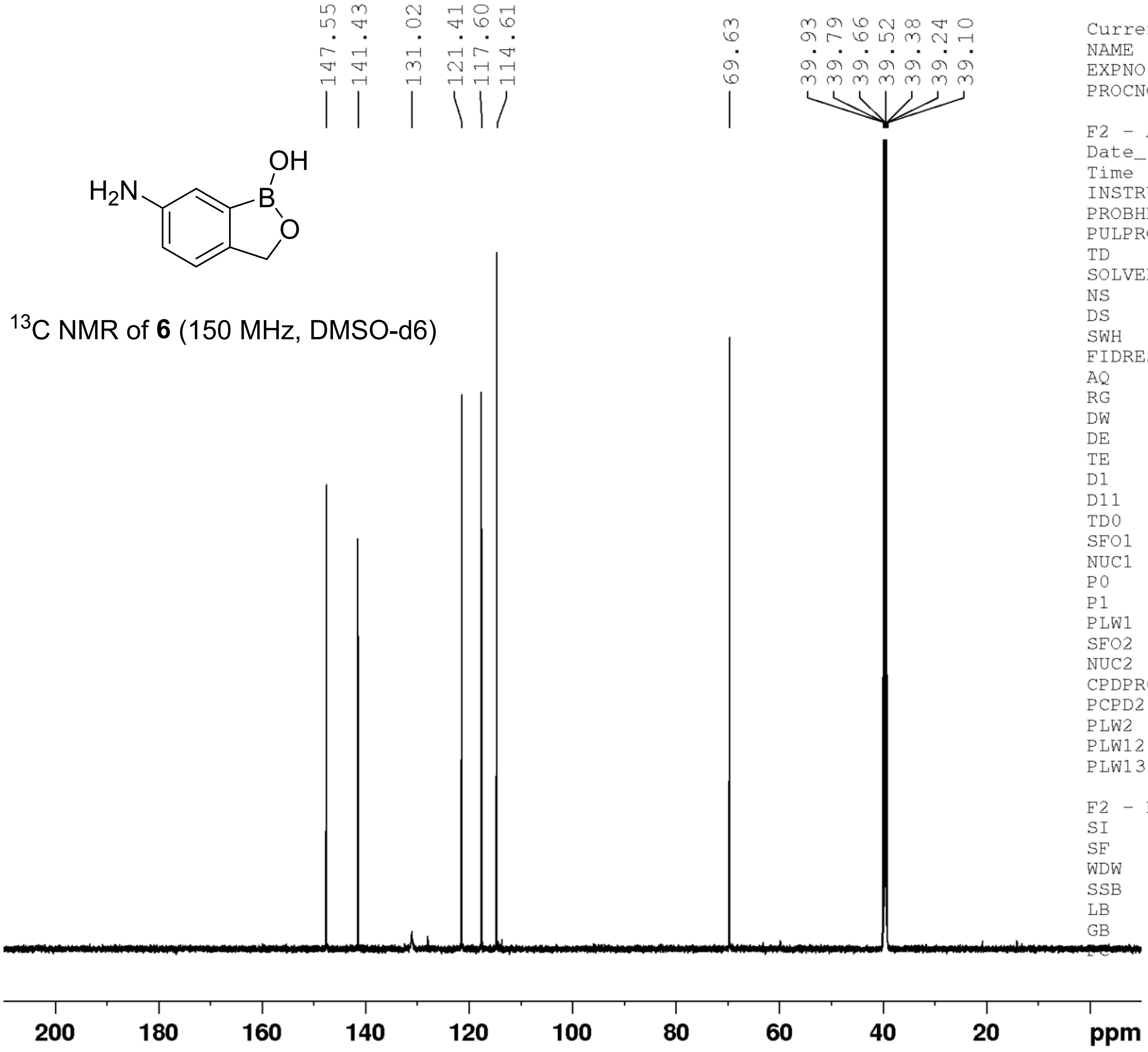

Current Data Parameters

NAME	PVK49-X78 A-5 Pure	
EXPNO	2	
PROCNO	1	

F2 - Acquisition Parameters

Date_	20230111	
Time	17.25	h
INSTRUM	spect	
PROBHD	Z148658_0003 (	
PULPROG	zgpg30	
TD	65536	
SOLVENT	DMSO	
NS	1024	
DS	4	
SWH	36231.883	Hz
FIDRES	1.105709	Hz
AQ	0.9043968	sec
RG	199.73	
DW	13.800	usec
DE	6.50	usec
TE	298.7	K
D1	2.00000000	sec
D11	0.03000000	sec
TD0	1	
SFO1	150.8864644	MHz
NUC1	13C	
P0	4.00	usec
P1	12.00	usec
PLW1	77.65699768	W
SFO2	600.0074000	MHz
NUC2	1H	
CPDPRG[2	waltz65	
PCPD2	70.00	usec
PLW2	13.23200035	W
PLW12	0.64876997	W
PLW13	0.32633001	W

F2 - Processing parameters

SI	32768	
SF	150.8714473	MHz
WDW	EM	
SSB	0	
LB	1.00	Hz
GB	0	
PC	1.40	

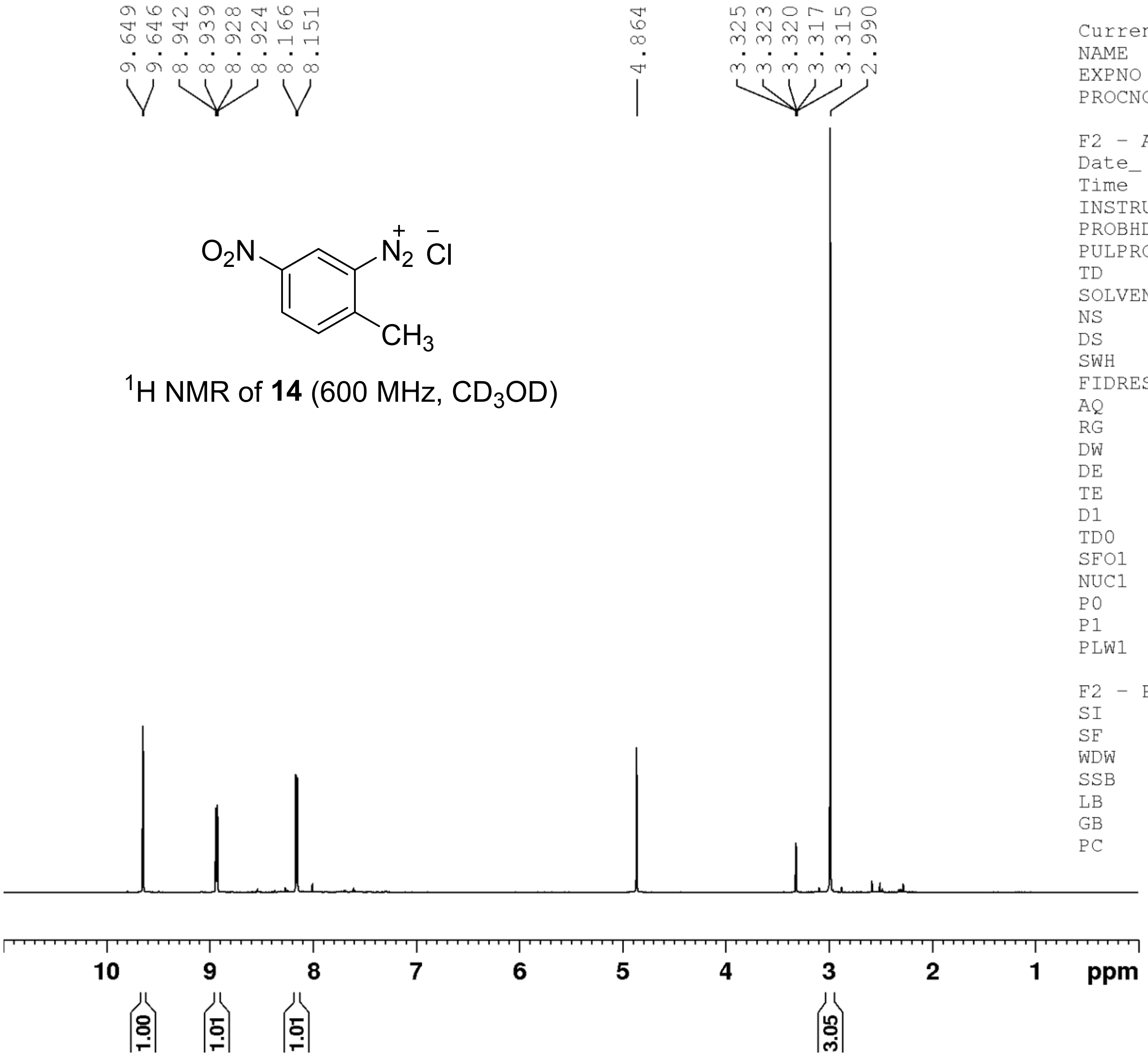

^{1}H NMR of **14** (600 MHz, CD_3OD)

```
Current Data Parameters
NAME     PVK49-X61 Diazo Salt Pure
EXPNO                    1
PROCNO                   1

F2 - Acquisition Parameters
Date_             20230110
Time                 17.12 h
INSTRUM               spect
PROBHD   Z148658_0003 (
PULPROG               zg30
TD                   65536
SOLVENT               MeOD
NS                      16
DS                       2
SWH              12019.230 Hz
FIDRES            0.366798 Hz
AQ               2.7262976 sec
RG                  157.38
DW                  41.600 usec
DE                   10.33 usec
TE                   296.6 K
D1              1.00000000 sec
TD0                      1
SFO1           600.0087050 MHz
NUC1                    1H
P0                    5.17 usec
P1                   15.50 usec
PLW1           13.23200035 W

F2 - Processing parameters
SI                   65536
SF             600.0050056 MHz
WDW                     EM
SSB                      0
LB                    0.30 Hz
GB                       0
PC                    1.00
```

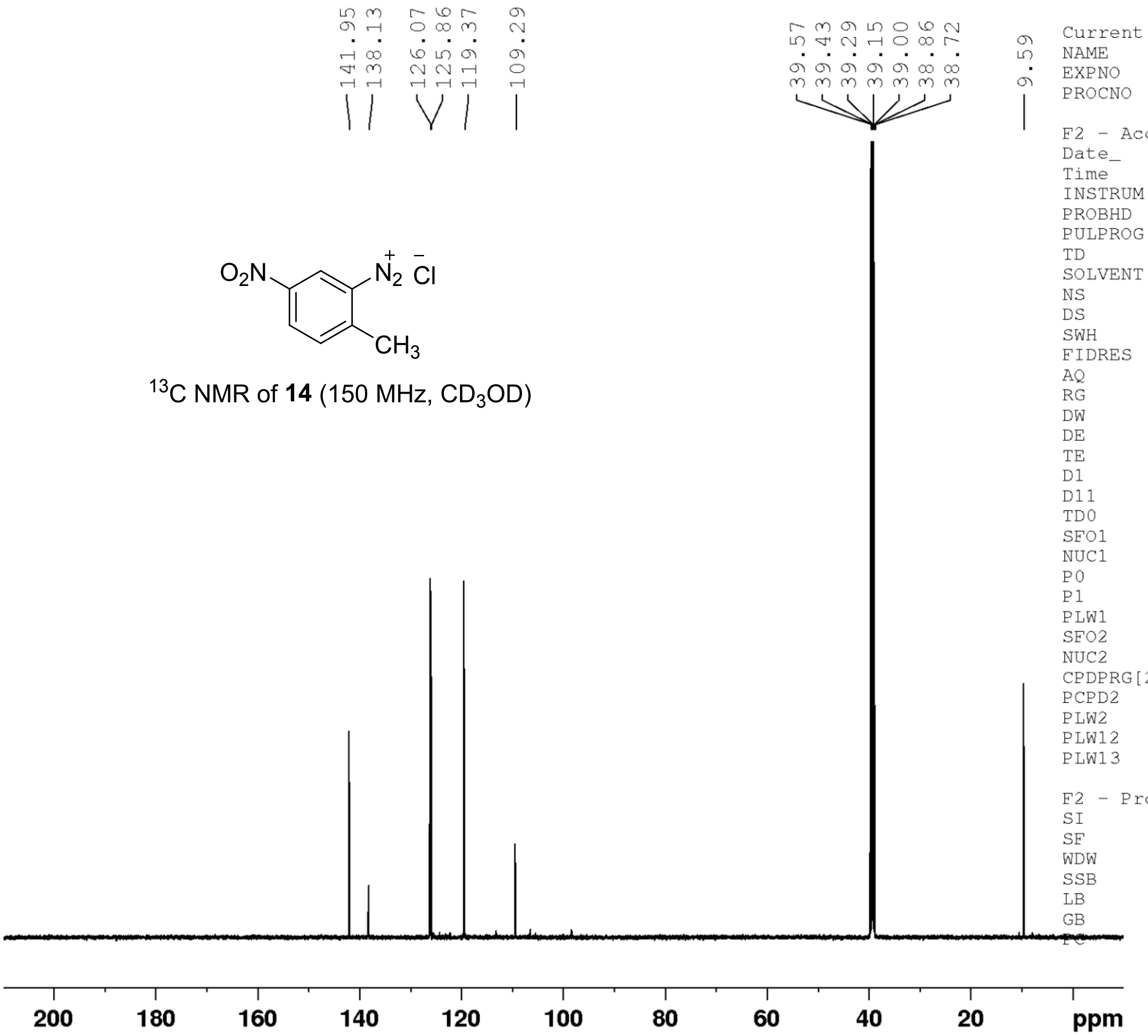

^{13}C NMR of **14** (150 MHz, CD_3OD)

Current Data Parameters
NAME PVK49-X61 Diazo Salt Pure
EXPNO 2
PROCNO 1

F2 - Acquisition Parameters
Date_ 20230110
Time 18.04 h
INSTRUM spect
PROBHD Z148658_0003 (
PULPROG zgpg30
TD 65536
SOLVENT MeOD
NS 1024
DS 4
SWH 36231.883 Hz
FIDRES 1.105709 Hz
AQ 0.9043968 sec
RG 199.73
DW 13.800 usec
DE 6.50 usec
TE 298.0 K
D1 2.00000000 sec
D11 0.03000000 sec
TD0 1
SFO1 150.8864644 MHz
NUC1 13C
P0 4.00 usec
P1 12.00 usec
PLW1 77.65699768 W
SFO2 600.0074000 MHz
NUC2 1H
CPDPRG[2 waltz65
PCPD2 70.00 usec
PLW2 13.23200035 W
PLW12 0.64876997 W
PLW13 0.32633001 W

F2 - Processing parameters
SI 32768
SF 150.8726596 MHz
WDW EM
SSB 0
LB 1.00 Hz
GB 0
PC 1.40

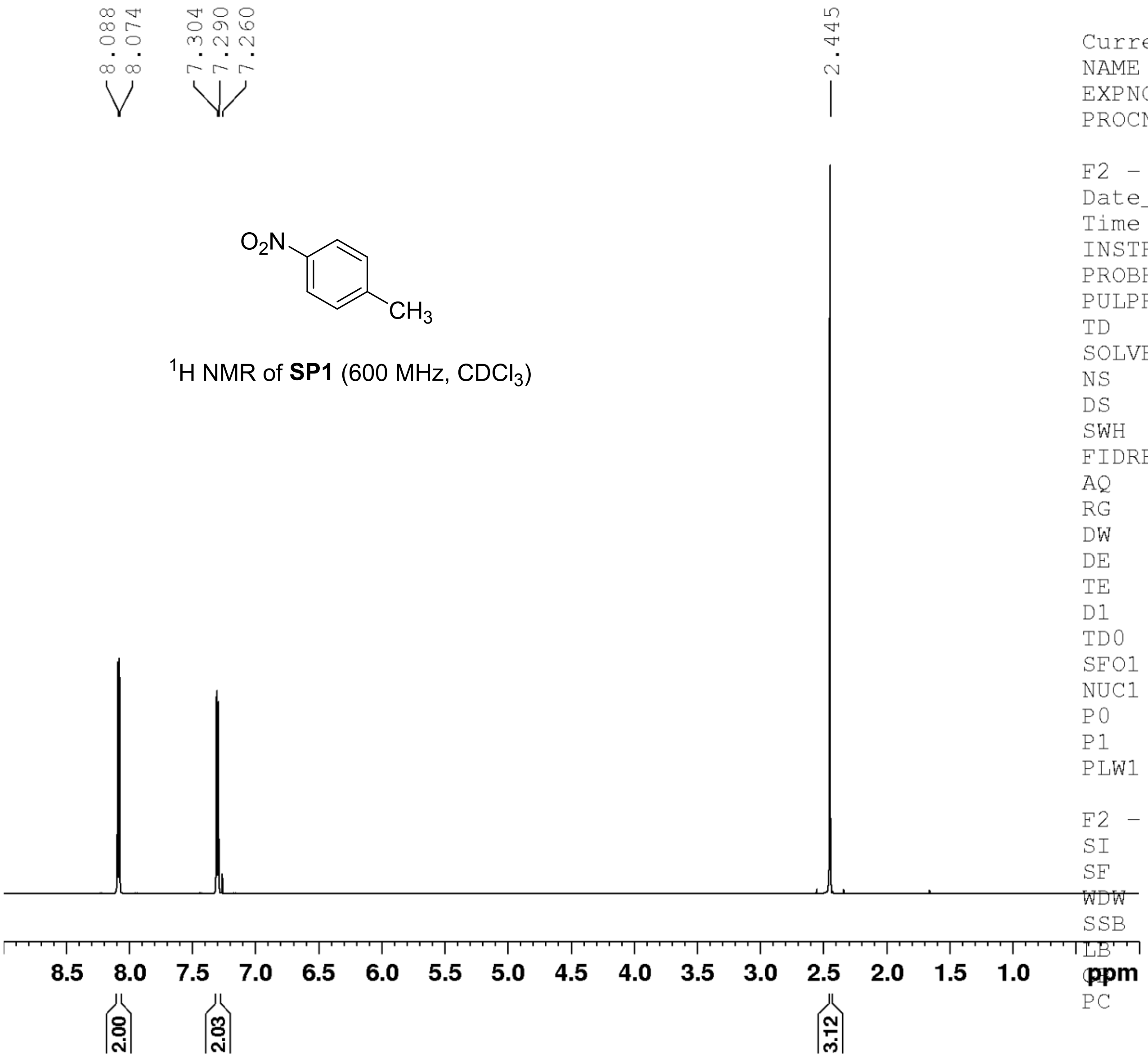

^1H NMR of **SP1** (600 MHz, $CDCl_3$)

Current Data Parameters
NAME PVK49-X8 S.M Pure
EXPNO 1
PROCNO 1

F2 - Acquisition Parameters
Date_ 20230106
Time 15.42 h
INSTRUM spect
PROBHD Z148658_0003 (
PULPROG zg30
TD 65536
SOLVENT CDCl3
NS 16
DS 2
SWH 12019.230 Hz
FIDRES 0.366798 Hz
AQ 2.7262976 sec
RG 55.05
DW 41.600 usec
DE 10.33 usec
TE 296.3 K
D1 1.00000000 sec
TD0 1
SFO1 600.0087050 MHz
NUC1 1H
P0 5.17 usec
P1 15.50 usec
PLW1 13.23200035 W

F2 - Processing parameters
SI 65536
SF 600.0050153 MHz
WDW EM
SSB 0
LB 0.30 Hz
GB 0
PC 1.00

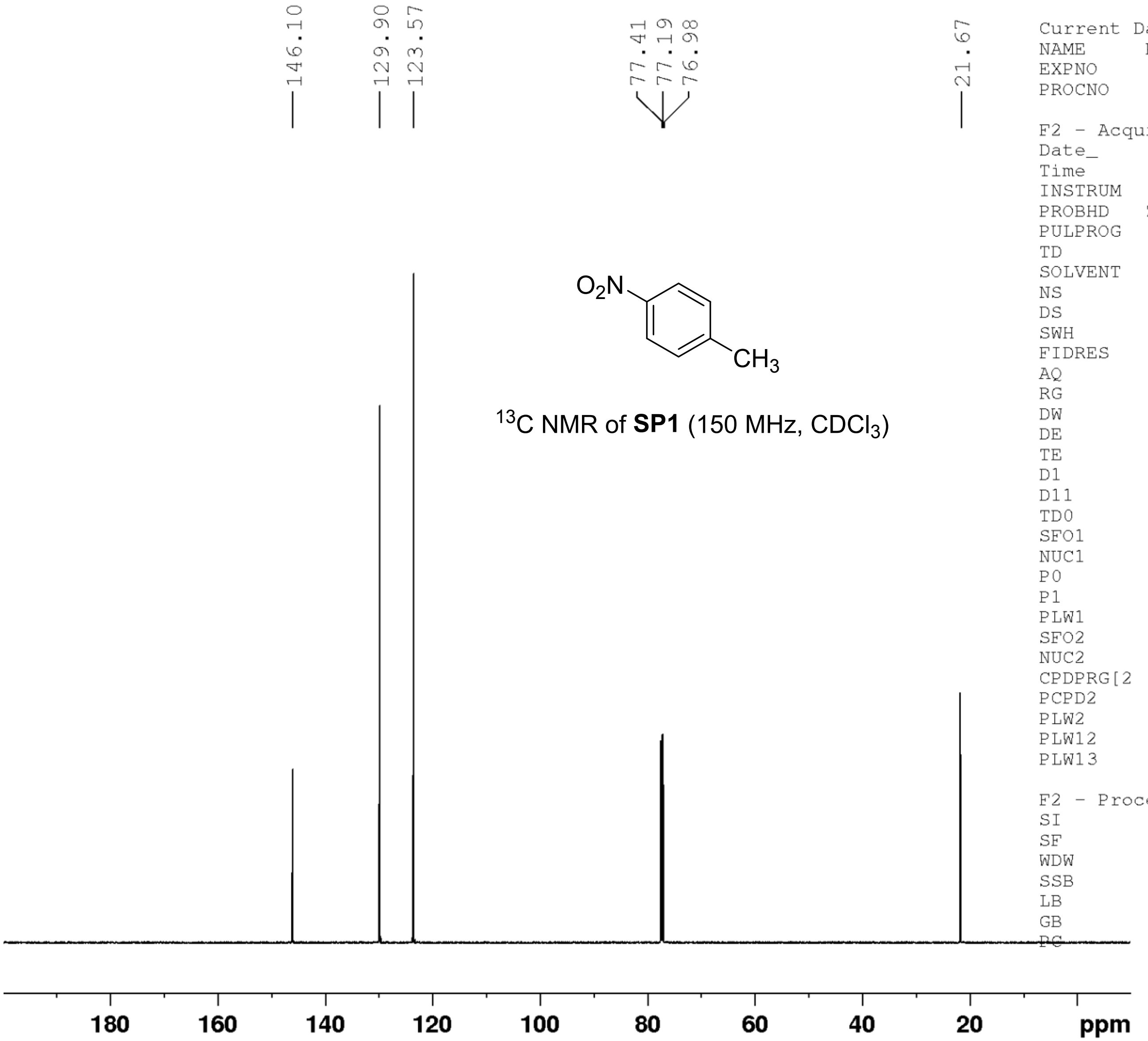

^{13}C NMR of **SP1** (150 MHz, $CDCl_3$)

Current Data Parameters
NAME PVK49-X8 S.M Pure
EXPNO 2
PROCNO 1

F2 - Acquisition Parameters
Date_ 20230106
Time 16.34 h
INSTRUM spect
PROBHD Z148658_0003 (
PULPROG zgpg30
TD 65536
SOLVENT CDCl3
NS 1024
DS 4
SWH 36231.883 Hz
FIDRES 1.105709 Hz
AQ 0.9043968 sec
RG 199.73
DW 13.800 usec
DE 6.50 usec
TE 298.3 K
D1 2.00000000 sec
D11 0.03000000 sec
TD0 1
SFO1 150.8864644 MHz
NUC1 13C
P0 4.00 usec
P1 12.00 usec
PLW1 77.65699768 W
SFO2 600.0074000 MHz
NUC2 1H
CPDPRG[2 waltz65
PCPD2 70.00 usec
PLW2 13.23200035 W
PLW12 0.64876997 W
PLW13 0.32633001 W

F2 - Processing parameters
SI 32768
SF 150.8713629 MHz
WDW EM
SSB 0
LB 1.00 Hz
GB 0
PC 1.40
